# Contrasting roles for G-quadruplexes in regulating human Bcl-2 and virus homologues KSHV KS-Bcl-2 and EBV BHRF1

**DOI:** 10.1038/s41598-022-08161-9

**Published:** 2022-03-23

**Authors:** Shivani Kumar, Chitteti Ramamurthy, Divya Choudhary, Aashika Sekar, Anupam Patra, Neel Sarovar Bhavesh, Perumal Vivekanandan

**Affiliations:** 1 Kusuma School of Biological Sciences, Indian Institute of Technology, Delhi, New Delhi 110016 India; 2 Department of Chemical Engineering, Indian Institute of Technology, Delhi, New Delhi 110016 India; 3 Department of Biochemical Engineering and Biotechnology, Indian Institute of Technology, Delhi, New Delhi 110016 India; 4 Transcription Regulation Group, International Centre for Genetic Engineering and Biotechnology, Delhi, New Delhi 110067 India

**Keywords:** Chemical biology, Molecular biology

## Abstract

Herpesviruses are known to acquire several genes from their hosts during evolution. We found that a significant proportion of virus homologues encoded by HSV-1, HSV-2, EBV and KSHV and their human counterparts contain G-quadruplex motifs in their promoters. We sought to understand the role of G-quadruplexes in the regulatory regions of viral Bcl-2 homologues encoded by KSHV (KS-Bcl-2) and EBV (BHRF1). We demonstrate that the KSHV KS-Bcl-2 and the EBV BHRF1 promoter G-quadruplex motifs (KSHV-GQ and EBV-GQ) form stable intramolecular G-quadruplexes. Ligand-mediated stabilization of KS-Bcl-2 and BHRF1 promoter G-quadruplexes significantly increased the promoter activity resulting in enhanced transcription of these viral Bcl-2 homologues. Mutations disrupting KSHV-GQ and EBV-GQ inhibit promoter activity and render the KS-Bcl-2 and the BHRF1 promoters non-responsive to G-quadruplex ligand. In contrast, promoter G-quadruplexes of human *bcl-2* gene inhibit promoter activity. Further, KS-Bcl-2 and BHRF1 promoter G-quadruplexes augment RTA (a virus-encoded transcription factor)-mediated increase in viral *bcl-2* promoter activity. In sum, this work highlights how human herpesviruses have evolved to exploit promoter G-quadruplexes to regulate virus homologues to counter their cellular counterparts.

## Introduction

Herpesviruses are ubiquitous double-stranded DNA (dsDNA) viruses. Alphaherpesvirinae, Betaherpesvirinae, and Gammaherpesvirinae are subfamilies of *Herpesviridae* that are delineated on the basis of cellular tropism, efficiency of viral replication and manifestation of viral disease^1–3^. Herpesviruses differ from other viruses in their ability to establish latency in the infected host^4–6^. During the course of evolution, herpesviruses have captured several host genes that facilitate their survival in the host^7–12^. Once acquired, the virus homologues of human genes perform a wide array of functions including evading host immune responses, regulating apoptosis in virus infected cells, and regulating the host transcription and translation machinery^13–18^. Four human gene homologues (i.e., DNA helicase, DNA-dependent DNA polymerase, Ribonucleotide reductase large subunit and, Uracil-DNA glycosylase) are known to be present in all human herpesviruses. A sizable proportion (~ 13%) of proteins encoded by human herpesviruses are homologues of human proteins^19^. About a third of all proteins encoded by Kaposi’s sarcoma associated herpesvirus (KSHV; Human Herpesvirus 8) share homology with human proteins^20–22^.

We hypothesized that while capturing host genes, human herpesviruses have also captured the regulatory regions of the host genes. In other words, we wanted to understand if virus homologues of cellular proteins encoded by human herpesviruses also mimic the regulatory regions of their human counterparts. In particular, we wanted to study the role of G-quadruplexes which are nucleic acid secondary structures in regulatory regions of virus homologues and their human counterparts.

G-quadruplexes are non-canonical nucleic acid secondary structures facilitated by hoogsteen hydrogen bonding. These structures are composed of three or more G-tetrads stacked upon each other. G-quadruplexes are enriched in promoters of human genes and they modulate the promoter activity of several proto-oncogenes including *c-myc, c-kit1, c-kit2, vegf, k-ras, met, hif1α* and *bcl-2*^23–32^.

Genomes of human herpesviruses are enriched for G-quadruplexes^33–36^. Studies have revealed the antiviral effect of G-quadruplex specific ligands^37–40^. These ligands were also found to interact with G-quadruplexes found in herpesvirus genomes and significantly alter virus life cycle^41–43^. G-quadruplexes in herpesvirus gene promoters modulate promoter activity and regulate gene expression^44–47^. Viral Bcl-2 (vBcl-2) represents one of the most well studied herpesvirus homologues of human proteins. Gammaherpesviruses infecting humans i.e., Kaposi’s sarcoma associated herpesvirus (KSHV/HHV8) and Epstein Barr Virus (EBV/HHV4) are known to encode for viral Bcl-2 homologues namely, KSHV KS-Bcl-2 protein (encoded by *orf16* gene) and EBV BHRF1 protein (encoded by the *bhrf1* gene)^16,48–50^. These viral Bcl-2 homologues are known to share homology with each other and also with their cellular counterpart^51^. The vBcl-2 proteins are expressed early during lytic replication and are known to regulate apoptosis of the infected host cells to promote release of viral progeny^48,51–53^.

We performed systematic screening to identify G-quadruplexes in virus homologues and their human counterparts. We identified putative quadruplex sequence (PQS) motifs in the regulatory regions (promoters) of virus homologues and their human counterparts. These PQS formed stable G-quadruplexes in vitro. To understand the biological role of these promoter G-quadruplexes in virus homologues we chose to study vBcl-2. We performed extensive functional characterization of G-quadruplexes in the promoters of KSHV KS-Bcl-2 and EBV BHRF1. We found that the G-quadruplex in both the promoters of KS-Bcl-2 and BHRF1 enhance viral Bcl-2 expression. On the contrary, the G-quadruplexes in human *bcl-2* gene promoter supress gene expression. Studies with G-quadruplex binding ligands and mutations disrupting the G-quadruplex in the KS-Bcl-2 and BHRF1 promoters confirm a positive regulatory role for these secondary structures. Further, we also demonstrate a role for these vBcl-2 promoter G-quadruplexes in facilitating virus transcription factor mediated enhancement of vBcl-2 expression. We found that human herpesvirus homologues often mimic regulatory regions of their human counterparts. Herpesviruses exploit these secondary structures in the promoters of virus homologues to their advantage through virus-encoded transcription factors.

## Materials and methods

### Retrieval of sequences

The upstream regulatory region (-5000 bp to + 100 bp) with respect to the transcription start site of human genes (n = 28) that are known to have homologues in human herpesviruses were retrieved from Eukaryotic Promoter Database (EPD) freely accessible at https://epd.epfl.ch//index.php^54^. Although only a small proportion of herpesvirus promoters have been experimentally validated, previous reports in literature suggest that herpesvirus promoters are typically found within the 1 kb upstream region of the genes^44,55–59^. Taking this into consideration, the 1 kb upstream regulatory region of viral homologues of human herpesviruses (HHV1, HHV2, HHV3, HHV4, HHV5, HHV6A, HHV7, HHV8) along with human adenoviruses (Classes A, B1, B2, C, D, E, F, G), African swine fever virus (ASFV), Alcelaphine herpesvirus 1 (AHV1), Bovine herpesvirus 4 (BoHV4), Murine gammaherpesvirus 68 (GH68), Meleagrid herpesvirus 1 (MHV1), Lymphocystis disease virus (LDV), Frog virus 3 (FV3), Singapore grouper iridovirus (SGIV), Fowlpox virus (FPV) and ORF virus (ORFV) were obtained from ViPR (http://www.viprbrc.org) database^60^ and NCBI GenBank (http://www.ncbi.nlm.nih.gov) for PQS mining. The 1 kb upstream regulatory regions are henceforth referred to as putative promoter regions of homologous genes. Accession number of virus strains used in the study is listed in Supplementary File 2: Table S2T1 and Table S2T2.

### PQS mapping and randomization

The retrieved upstream sequences (human and viral) were analysed to identify PQS motifs with parameters (minimum G-tetrad = 3 and loop length = 1–15) using Quadparser^61^. PQS density was defined as the total number of non-overlapping PQS predicted per kilo base of the sequence analysed. The selected sequences were then shuffled while preserving their individual dinucleotide frequencies to determine whether the presence of PQS motifs is a random event. A python program script^62^ based on the freely available ‘uShuffle’ program script^63^ was used for dinucleotide shuffling of the 1 kb upstream regulatory sequences of human herpesvirus Bcl-2 homologues (i.e., KS-Bcl-2 and BHRF1). Average PQS densities were calculated in the randomized sequences generated and were compared to that in the native sequences.

### PQS conservation analyses

Upstream regulatory regions with at least 1 PQS motif were analysed for conservation. Accession numbers of all sequences analysed are provided in Supplementary File 2; Table S2T2. A conserved PQS motif does not necessarily possess identical core guanine (G) residues. PQS motifs with adjacent Gs capable of forming a quadruplex were also deemed as conserved.

### CD spectroscopy and CD melting analyses

CD spectroscopy was performed using oligonucleotides corresponding to the randomly selected PQS in the promoter regions of human herpesvirus homologues (n = 15) and their cellular counterparts (n = 9). The sequences of the selected PQS oligonucleotides are provided in Supplementary File 2; Table S2T3 and Table S2T4. All synthetic oligonucleotides were purchased from Integrated DNA technologies (IDT) for spectroscopic analyses. Oligonucleotide samples were mixed at a concentration of 10 µM in 10 mM sodium cacodylate buffer (pH -7.5) containing 100 mM KCl. The prepared samples were heated at 95 °C for 5 min and then gradually cooled to room temperature. PQS oligonucleotides from upstream regulatory regions of KSHV KS-Bcl-2 and EBV BHRF1 genes (Wt-KSHV-GQ and Wt-EBV-GQ oligonucleotides along with their corresponding mutants Mut-KSHV-GQ and Mut-EBV-GQ) were prepared in a similar manner (Sequences are provided in Supplementary File 2, Table S2T5). In addition, CD melting was performed using Wt-KSHV-GQ and Wt-EBV-GQ PQS oligonucleotides in the presence and absence of G-quadruplex ligand pyridostatin (PDS; 5 µM). Data were recorded at a ramp rate of 1 °C/minute over a range of 20–93 °C at a fixed wavelength of 262 nm. T_m_ was calculated using the first derivative method.

### UV melting analyses

UV melting experiments were performed using Cary 100 Bio UV–Vis double-beam spectrophotometer (Agilent Technologies) equipped with a multi-cell holder attached to a Peltier controller. The Wt-KSHV-GQ and Wt-EBV-GQ oligonucleotides (4 µM) were dissolved in 10 mM sodium cacodylate buffer (pH -7.5) and 100 mM KCl. The samples were then heated to 95 °C for 5 min and gradually cooled to room temperature. Pyridostatin at a concentration of 5 µM was used for melting analysis. The melting curves were recorded at 295 nm between 20 °C and 95 °C with a ramp rate of 1 °C/min. Origin 9.0 (Origin Lab Corp.) was used to analyse and plot melting curves. T_m_ was calculated using the first derivative method.

### Polyacrylamide gel electrophoresis

Samples were prepared using PQS oligonucleotides (Wt-KSHV-GQ and Wt-EBV-GQ and their corresponding mutants with length matched controls; Supplementary File 2; Table S2T5) at 10 µM concentration in Tris–EDTA buffer (pH -7.0) and 100 mM KCl. The prepared samples were subjected to heating at 95 °C for 5 min followed by slow cooling to room temperature before loading. Native and denaturing polyacrylamide gels were prepared in 1 × Tris–borate EDTA (TBE) buffer. The denaturing polyacrylamide gel was supplemented with 7 M urea as a denaturant. Both gels were run in 0.5 × TBE with 50 mM KCl. Full length images are available in Supplementary File 3.

### NMR spectroscopy

Samples for NMR spectroscopy were prepared using PQS oligonucleotides (Wt-KSHV-GQ and Wt-EBV-GQ and their corresponding mutants; Supplementary File 2; Table S2T5) at a concentration of 300 μM in 20 mM phosphate buffer (pH -7.0) supplemented with 100 mM KCl and 10% D_2_O (*v/v*). The samples were then heated to 95 °C and allowed to cool slowly to room temperature. 1D ^1^H NMR spectra were recorded at a constant temperature of 20 °C on a Bruker Avance III spectrometer equipped with cryogenic 5 mm TCI triple-resonance probe, operating at a field strength of 500 MHz. Topspin 3.5 (Bruker AG) was used for data acquisition. Data processing and plotting of spectra were done using Topspin 4.6 software (Bruker AG).

### Dimethyl sulphate (DMS) footprinting

Synthetic 5’ FAM labelled PQS oligonucleotides were purchased from Integrated DNA Technologies (IDT) for footprinting analyses (Supplementary File 2; Table S2T6). The oligonucleotides were prepared in Tris–EDTA (10 mM) with and without KCl, heated to 95 °C followed by gradual cooling to room temperature to allow G-quadruplex formation. These oligonucleotides were further treated with 0.5% DMS for 2 min to allow methylation of guanine residues. This reaction was stopped using 1 μg of calf thymus DNA. The reactions were run on a 20% native polyacrylamide gel. The desired samples were gel extracted and subjected to ethanol precipitation. The extracted samples were then cleaved using 1 M piperidine at 95 °C. Cleaved products were dried using a SpeedVac machine, and further re-suspended in alkaline sequencing dye. The final products were then resolved on a 20% denaturing polyacrylamide gel. Full length images are available in Supplementary File 3.

### Cloning of KSHV KS-Bcl-2 and EBV BHRF1 promoter regions

The PQS containing 1 kb promoter region of KSHV KS-Bcl-2 was amplified using HHV8 DNA (JSC-1; courtesy Dr. Tathagata Choudhuri, Visva Bharati University, Bolpur, India) and the PQS containing 1 kb promoter region of EBV BHRF1 was commercially synthesized by Life Technologies Corp. The amplified products were cloned into a promoter less firefly luciferase vector (pGL3 basic vector; Promega).

### Cloning of KSHV KS-Bcl-2 and EBV BHRF1 coding regions with their native promoters

For mRNA quantification studies, the KSHV KS-Bcl-2 protein coding region was cloned along with its 1 kb promoter in ∆luc pGL3 basic vector. Similarly, the EBV BHRF1 protein coding region was also subcloned from the MSCV-N BHRF1 construct {MSCV-N BHRF1 was a gift from Dr. Karl Munger (Addgene plasmid #37,937; http://n2t.net/addgene:37937;RRID:Addgene_37937)^64^ along with its 1 kb promoter in ∆luc pGL3 basic vector. Mutations were incorporated into PQS containing regions to disrupt G-quadruplex formation. Sequences of mutant oligonucleotides are provided in Fig. 3a. The primers used for amplification and cloning (wild type and mutants) are provided in Supplementary File 2; Table S2T7. The plasmid constructs were purified using QIAprep Spin Miniprep Kit (Qiagen) and confirmed by sequencing. The human *bcl-2* gene promoter luciferase construct LB322 (Bcl-2 from ATG to -3934) was a gift from Dr. Linda Boxer (Addgene plasmid #15,381; http://n2t.net/addgene:15381;RRID:Addgene_1538)^65^.

### Cell lines

HEK293T cells were procured from NCCS, Pune, India and were maintained in Dulbecco’s modified medium (Invitrogen) supplemented with 10% fetal bovine serum (Invitrogen) and incubated at 37 °C with 5% CO_2_.

### Luciferase reporter assay

For promoter activity analyses, HEK293T cells were seeded in a 24-well plate at a concentration of 5 × 10^4^ cells and co-transfected with luciferase constructs containing KS-Bcl-2 and BHRF1 promoters (wild type or mutant) along with pRL-TK vector (a renilla luciferase reporter construct with a thymidine kinase promoter) at the concentration ratio of 25:1 (i.e., 500 ng for pGL3 constructs and 20 ng for pRL-TK) using PEI (polyethylenimine). To determine the effect of ligand on promoter activity, 5 µM pyridostatin (PDS) was added to the samples 2 h after transfection^62^. Additionally, we co-transfected KSHV-RTA (Plasmid pcDNA-RTA;100 ng) and EBV-RTA (Plasmid pSG5-BRLF1;100 ng) with the KSHV KS-Bcl-2 and EBV BHRF1 promoter constructs, respectively, to understand the role of viral RTA in regulating the promoter activity of these virus homologues. Plasmid pcDNA-RTA was a kind gift from Dr. Erle S. Robertson (University of Pennsylvania, Philadelphia, PA, USA)^66^. Plasmid pSG5-BRLF1 was a gift from Dr. S. Diane Hayward (Addgene plasmid #72635; http://n2t.net/addgene:72635;RRID:Addgene_72635)^67^. Cell lysates were obtained at 24 h post transfection. Luciferase assay was carried out using a dual luciferase reporter assay system (Promega) according to the manufacturer’s protocol. Luminescence values for both firefly and renilla were recorded using a MicroBeta2 Microplate Scintillation Counter (Perkin Elmer). Firefly luciferase activity was normalized to Renilla luciferase activity. Three independent experiments were done in triplicates.

### Quantitative real time PCR

Total RNA was extracted from transfected HEK293T cells using TRIzol reagent (ThermoFisher scientific) according to the manufacturer's protocol. The extracted RNA (500 ng) was treated with 1 μg of DNase I (New England Biolabs) to remove residual DNA. cDNA was synthesized using iScript cDNA synthesis kit (Biorad). Real time PCR was performed using the FastStart essential DNA green master mix (Roche) for quantifying viral Bcl-2 mRNA levels i.e., KSHV KS-Bcl-2 and EBV BHRF1 mRNA levels. The appropriate primers used for qPCR studies are listed in Supplementary File 2; Table S2T8. Glyceraldehyde 3-phosphate dehydrogenase (GAPDH) was used as an internal control for quantification. Fold change in expression was calculated using the 2^-∆∆Ct^ method^68^.

### Data analyses, graphical representation, and statistics

Microsoft Excel was used for analysis of data and plotting of bar graphs unless mentioned otherwise. Origin 9.0 was used for plotting CD curves. Figure 7 was created using Microsoft PowerPoint. Student’s t-test was used to determine statistical significance. P values less than 0.05 were considered significant.

## Results

### Abundance of PQS motifs in promoters of herpesvirus homologues and their human counterparts

**Figure 1:**
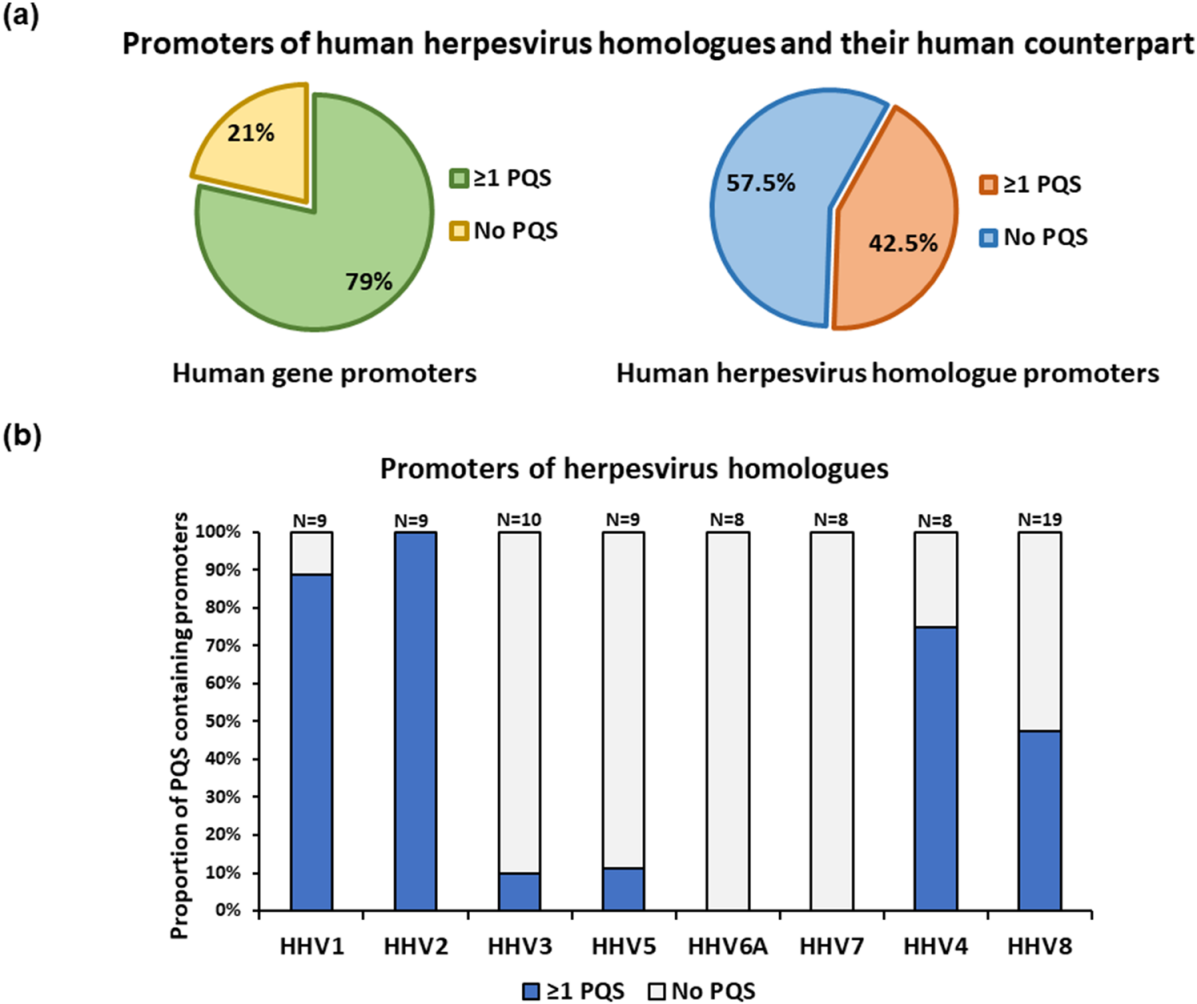
A significant proportion of promoters of human genes captured by herpesviruses and the corresponding virus homologues contain at least one PQS motif. (**a**) Pie charts show the proportion of promoters of human genes (~ 79%) captured by herpesviruses and that of corresponding herpesvirus homologues (~ 42%) with at least 1 PQS motif. (**b**) Bar graph shows the percentage of viral homologues from human herpesviruses with at least 1 PQS motif in their upstream regulatory regions. Promoters of virus homologues from HHV1, HHV2 and gammaherpes viruses (HHV4 and HHV8) were enriched for PQS motifs. *N* denotes number of virus homologues.

We analysed human genes (n = 28) that are reported to share sequence homology with several herpesvirus genes^19^, for the presence of PQS motifs in their upstream regulatory regions (-5000 bp to + 100 bp)^69^ using Quadparser^61^. It was observed that 79% of these human genes possess at least 1 PQS motif in their upstream regulatory region (coding strand and/or template strand) (Fig. 1a). Additional details such as Ensemble ID, length, orientation of the human genes used for analyses is provided in Supplementary File 1, Table S1T1. Correspondingly, we also analysed the 1 kb upstream regulatory regions of the homologous genes encoded by human herpesviruses for PQS motifs. Although, minimal promoter regions (< 1 kb upstream region) have been described for some herpesvirus genes^55,70–72^, we performed PQS mining using a consensus sequence for the complete 1 kb putative promoter regions or the upstream regulatory regions. Over 40% of human herpesvirus homologues were found to possess at least 1 PQS motif in their upstream regulatory regions (Fig. 1a). The promoters of homologues encoded by human herpesvirus 1 (HHV1/HSV-1; Herpes simplex virus 1), human herpesvirus 2 (HHV2/HSV-2; Herpes simplex virus 2), human herpesvirus 4 (HHV4/EBV; Epstein Barr Virus) and human herpesvirus 8 (HHV8/KSHV; Kaposi’s sarcoma-associated herpesvirus) were particularly enriched for PQS motifs. In contrast, majority of promoters of homologues encoded by human herpesvirus 3 (HHV3/VZV; Varicella zoster virus) and betaherpesviruses [human herpesvirus 5 (HHV5/HCMV; Human cytomegalovirus), human herpesvirus 6A (HHV-6A) and human herpesvirus 7 (HHV-7)] did not contain PQS motifs (Fig. 1b).

Notably, all alpha and gamma human herpesviruses (HHV1, HHV2, HHV3, HHV4, HHV8) encode for corresponding viral homologues of five human genes in particular (with PQS containing promoters) i.e., DNA polymerase (POLA), DNA helicase (HFM1), Uracil DNA glycosylase (UNG1), Ribonucleotide reductase large subunit (RRM1) and Ribonucleotide reductase small subunit (RRM2)^19^. However, it was noted that only homologues encoded by HHV1 and HHV2 for all five genes (POLA, HFM1, UNG1, RRM1 and RRM2) contained at least 1 PQS in their upstream 1 kb regulatory regions (Table 1). Overall, it was observed that the majority of PQS containing human gene promoters (16 out of 22) do have corresponding human herpesvirus homologues (n = 29) with each possessing at least 1 PQS motif in their promoter (Supplementary File 1; Table S 1T1 and Figure S1F1). The PQS motifs found in herpesvirus putative promoter regions are comprehensively described in Supplementary File 2; Table S2T9.

**Table 1:** PQS motifs in promoters of h uman herpesvirus homologues belonging to different functional classes.

Gene class function	Human genes	Human herpesvirus genes							
		Alpha			Beta			Gamma	
		1	2	3	5	6A	7	4	8
DNA replication	DNA polymerase alpha (POLA)	**UL30**	**UL30**	ORF28	UL54	U38	U38	BALFS	ORF9
	DNA helicase (HFMl )	**UL5**	**UL5**	ORF55	UL1O5	U77	U77	BBLF4	ORF40
Nucleotide repair & metabolism	Uracil DNA glycosylase (UNG1)	**UL2**	**UL2**	ORF59	UL114	U81	U81	**BKRF3**	ORF46
	Ribonucleotide reductase M1 (RRM1)	**UL39**	**UL39**	ORF19	UL45	U28	U28	**BORF2**	ORF61
	Ribonucleotide reductase M2 polypeptide (RRM2)	**UL40**	**UL40**	ORF18				**BaRFl**	ORF60
	Thymidylate synthase (TYMS)			ORF13					ORF70
	Dihydrofolate reductase (DHFR)								ORF2
Enzyme	Protein kinase cdc2-related PICTAIRE-2 (PCTK2)	UL3	**UL3**	ORF58	UL83	U54	U54		ORF36
	Serine/threonine-protein kinase PRP4 (PRP4)	**US3**	**US3**	ORF66					
Gene expression regulation	Ring finger protein (C3H2C3 type) 6 (RFP)	**ICPO (RL2)**	**ICPO (RL2)**	**ORF61**					
Glycoprotein	OX-2 membrane glycoprotein precursor (OX-2)					U85	U85		K14
Host-virus interaction	Flap structure-specific endonuclease 1 (FEN1)	**UL41**	**UL41**	ORF17					
	chemokine (C–C motif) receptor 2 (CKR2)				**UL33, US28**	US1	US1		
	G protein-coupled receptor 50 (GPR50)					U12	U12		ORF74
	Tumor necrosis factor receptor superfamily member 14 (TNFRSF14)				UL144				
	Small inducible cytokine subf.B, member 9B (IP-9)				UL47				
	Small inducible cytokine subf.A, member 26 (TSC-1)								K4.1
	Major histocompatibility complex, Class I,E (HLA1-E)				UL18				
	lnterleukin 10 (IL10)							**BCRFl**	
	B-cell lymphoma protein 2 (Bcl-2)							**BHRFl, BALFl**	**ORF16**
	CD80 anti gen (CD80)							**BARFl**	
	Interferon consensus seq.binding protein 1 (ICSBP1)								**viRF1**
	Interferon regulatory protein 4 (IRF4)								**viRF3**
	lnterleukin 6 (IL6)								**K2**
	Decay accelerating factor for complement (DAF)								**ORF4**
	Cyclin D1 (CCND1)								**ORF72**
	Major histocompatibility complex, Class I (HLA1)								**K3,KS**
	CASPS and FADD-Iike apoptosis regulator (FLIP)								ORF71

All human herpesvirus gene homologues of human genes were broadly classified into 6 functional groups based on gene function. The viral genes with ≥ 1 PQS motif in their 1 kb upstream regulatory region (promoter region) are represented in bold font.

### PQS motifs form G-qua druplex structures in vitro

CD s pectroscopic ana lyses was performed to determine the topology of randomly sele cted PQS motifs (n = 24) from promoters of human genes and their herpesvirus homologues. Details of the PQS oligonucleotides are provided in Supplementary File 2; Table S2T3 and S 2T4. These randomly selected PQS motifs were selected from the group of PQS containing promoters of human genes that match with their PQS cont aining promot ers of herpesvirus homologues (Supplementary File 1; Figure S1F1). Parallel G-quadruplexes exhibit a characteristic positive peak at 260 nm and a characteristic negative peak at 240 nm while antiparallel G-quadruplexes show a characteristic positive peak at 290 nm and a characteristic negative peak at 260 nm during CD spectroscopic analyses. Hybrid or mix-type G-quadruplexes exhibit distinct positive peaks, one each at 260 nm and 290 nm, and a negative peak at 240 nm. The CD spectra of randomly selected PQS motifs found in human gene prom oters (n = 9) and in the promoters of virus homologues (n = 15) show formation of parallel or hybrid G-quadruplexes (Supplementary File 1; Figure S1F2 and S1F3).

### PQS motifs in upstream regulatory re gions of viral Bcl-2 homologues are conserved

Viral Bcl-2 (vBcl-2) is among the most extensively studied homologues encoded by herpesviruses. Therefore, to investigate the functional role of G-quadruplex in promoters of virus homologues we chose to study viral *bcl-2* gene promoters. The human *bcl-2* gene is a well-studied example of a proto-oncogene reported to contain stable G-quadruplexes that supress Bcl-2 expression (26, 27, 32, 72, 73). The human Bcl-2 is a critical mitochondrial membrane protein that is known to regulate the intrinsic apoptotic pathway by mediating mitochondrial cytochrome c release upon apoptotic induction thereby acting as an apoptotic inhibitor^73,74^. Apart from herpesviruses, some adenoviruses and iridoviruses also encode for structurally and functionally similar Bcl-2 homologues^75–78^. Although this work is primarily focused on human herpesviruses and since we chose to study vBcl-2 in detail, we also wanted to analyse all known viral Bcl-2 homologues (BCL2DB database; http://bcl2db.ibcp.fr/).^79^ for PQS motifs in their upstream 1 kb regulatory regions. Of the 15 vBcl-2 homologues known till date, 9 of them contained a PQS motif in their 1 kb upstream regulatory region (Supplementary File 1; Table S1T2). For conservation analysis, we used all available full-length sequences from viruses with a PQS motif in their promoter. Our analysis suggests that the PQS motifs in 8 vBcl-2 promoters were highly conserved (Supplementary File 1; Figure S1F4). The presence of highly conserved PQS motifs in the promoters of vBcl-2 from herpesviruses and other viruses suggests a potential functional role for these DNA secondary structures across virus families.

**Figure 2:**
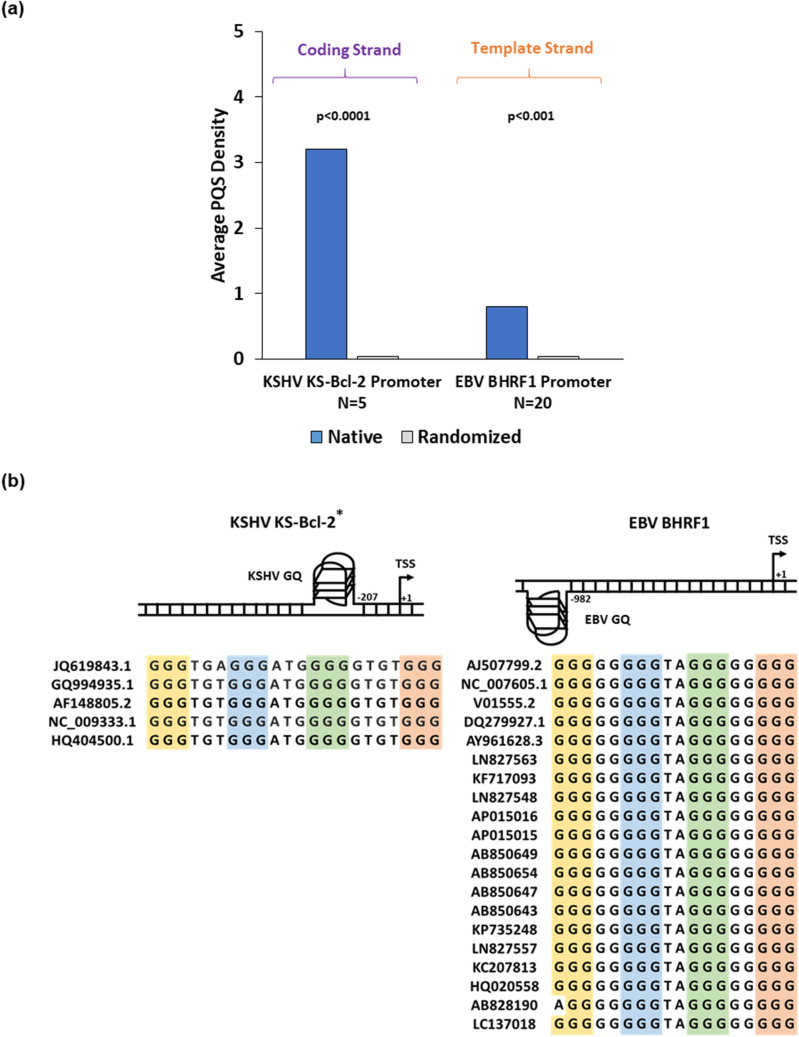
PQS motifs in regulatory regions of viral Bcl-2 homologues encoded by KSHV and EBV. (**a**) Bar graph shows PQS density in the 1 kb upstream regulatory region of KSHV KS-Bcl-2 and EBV BHRF1 genes. The PQS densities in the native sequences were significantly higher than that in the randomized sequences (shuffled sequences with constant dinucleotide content; detailed in "Materials and methods" section) (p < 0.001). (**b**) Schematic representation of the G-quadruplex motif closest to the TSS in KS-Bcl-2 and BHRF1 promoters. We refer to this G-quadruplex motif in KS-Bcl-2 and EBV BHRF1 as KSHV-GQ and EBV-GQ. Analysis of all full-length genomes from KSHV and EBV suggest that the KSHV-GQ and the EBV GQ are hi ghly conserved. *KS-Bcl-2 gene promoter (1 kb) was found to contain 3 PQS motifs at -207 nt., -255 nt., and -288 nt. from the TSS. Only the PQS motif closest to the TSS (at − 207 nt.) is represented in the schematic.

Among human herpesviruses, gammaherpesviruses KSHV *orf16* and EBV *bhrf1* genes encode for anti-apoptotic viral Bcl-2 proteins (KS-Bcl-2 and BHRF1 respectively) that share sequence and functional homology with the human *bcl-2* gene^48,51,80,81^. Previous studies reported that P1 and P2 promoters (as well as the upstream proximal promoter regions) of human *bcl-2* gene are enriched for G-quadruplexes and supress transcription of the human *bcl-2* gene^26,27,32,82^. In this study, we observed that KS-Bcl-2 and BHRF1 genes possess ≥ 1 PQS motifs in their upstream 1 kb regulatory regions. To verify whether the high PQS densities in KSHV and EBV *bcl-2* gene promoters is a random event or a consequence of their nucleotide composition, we randomized the upstream regulatory regions five times without changing the dinucleotide content of these sequences as described in “Mater ials and Methods” section. We found that the PQS densities in the native KSHV KS-Bcl-2 and EBV BHRF1 promoters were several fold higher than that of the respective randomized sequences (Fig. 2a); thus, indicating that the observed higher PQS density is not a random occurrence and is independent of the nucleotide composition of the primary sequence. We also observed that the upstream 1 kb regulatory region of KS-Bcl-2 gene contains multiple PQS species. As reported in previous genome-wide studies, GQs are predominant near the TSS in promoter regions of the human genome as well as viral genomes^45,69–88^. A large proportion of promoter GQs proximal to the TSS have strong implications in gene regulatory functions and are proven to be biologically relevant^89–91^. Hence, the PQS motif closest to the TSS of KS-Bcl-2 gene was chosen for further biophysical and functional analyses; this PQS motif is henceforth referred to as KSHV-GQ (Fig. 2). The EBV BHRF1 promoter contained only 1 PQS motif and this motif is henceforth referred to as EBV-GQ (Fig. 2). We also noticed that the selected PQS motifs in KS-Bcl-2 and BHRF1 promoters were highly conserved in all available full-length sequences (Fig. 2b).

### Biophysical characterization of GQ motifs upstream of KSHV KS-Bcl-2 and EBV BHRF1 genes

**Figure 3:**
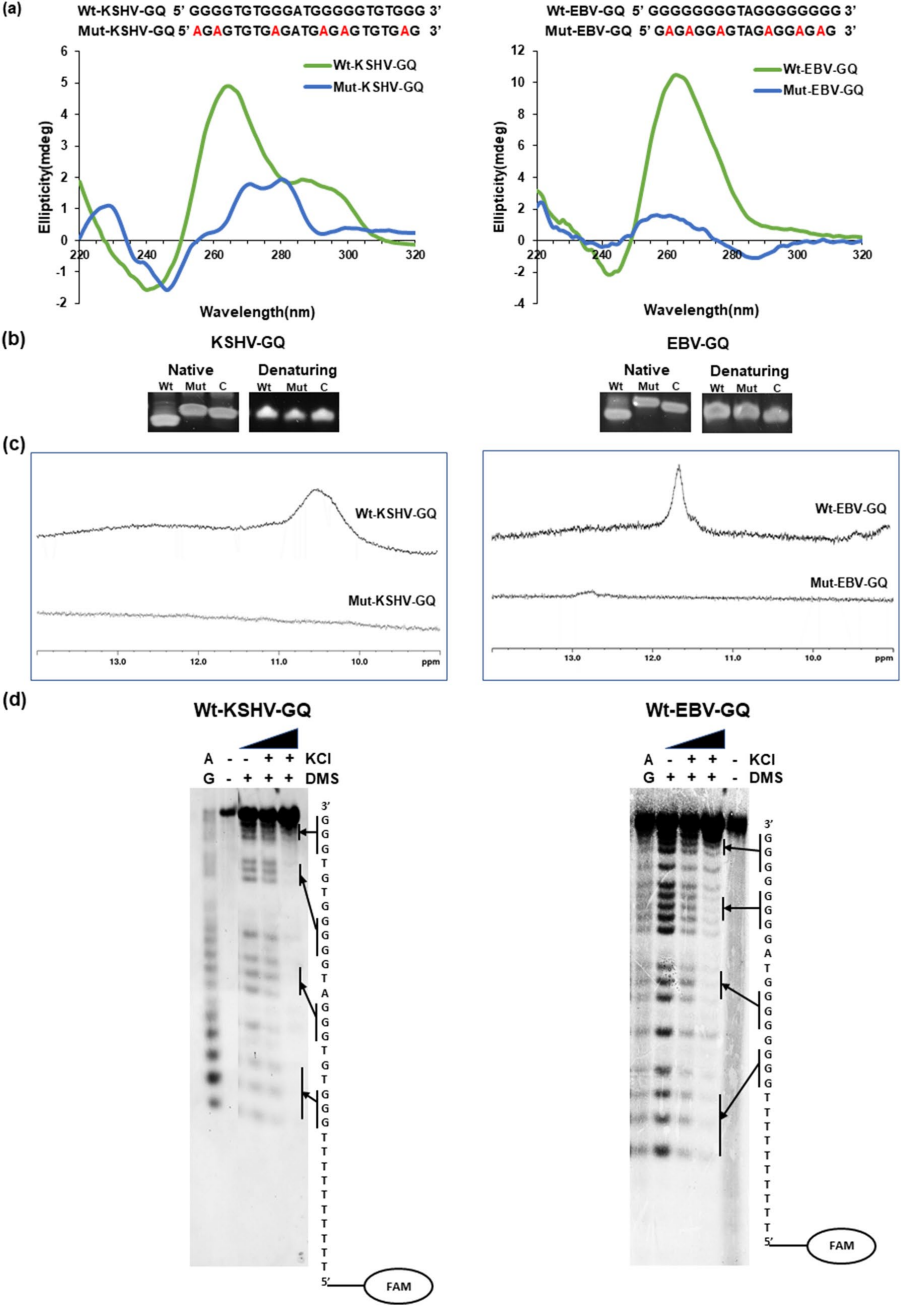
Biophysical analyses of KSHV-GQ and EBV-GQ reveals formation of stable intramolecular G-quadruplex in vitro. (**a**) CD spectra of Wt-KSHV-GQ and Wt-EBV-GQ show formation of hybrid (mixed type) and parallel G-quadruplex structures, respectively. Mutations were introduced to disrupt the G-quadruplex (Mut-KSHV-GQ and Mut-EBV-GQ). As expected, the mutant oligonucleotides did not form G-quadruplexes. (**b**) Native and denaturing polyacrylamide gel electrophoresis experiments reveal formation of intramolecular secondary structures attributed to the higher mobility of wild type oligonucleotides i.e., Wt-KSHV-GQ and Wt-EBV-GQ when compared with that of mutants (Mut-KSHV-GQ and Mut-EBV-GQ) and length match controls that do not form DNA secondary structures. (**c**) 1D ^1^H NMR spectra of Wt-KSHV-GQ and Wt-EBV-GQ oligonucleotide show resonance peaks of imino protons between 10.5–12 ppm, confirming the formation of G-quadruplexes. The NMR spectra suggest that Mut-KSHV-GQ and Mut-EBV-GQ do not form G-quadruplexes. (**d**) DMS footprinting experiments using 5’ FAM labelled oligonucleotides Wt-KSHV-GQ and Wt-EBV-GQ show formation of intramolecular G-quadruplex structures. Footprinting experiments were performed using different KCl concentrations {500 mM, 100 mM, and 0 mM (i.e., no KCl)}. AG represents seq uencing cleavage reactions specific to purine residues. The vertical bars next to the sequence of the oligonucleotide represents the G residues protected from DMS methylation due to formation of G-quadruplex structure. It is to be noted that the ladder (AG cleavage reaction lane) of the footprinting gel of Wt-KSHV-GQ has been snipped off from another part of the same gel and aligned together with the quadruplex sequence cleavage reaction lanes for comparison.

CD spectroscopic analysis using wild type (Wt) GQs i.e., Wt-KSHV-GQ and Wt-EBV-GQ reveal formation of a hybrid (mix-type) and a parallel G-quadruplex structure, respectively. G to A mutations in the Wt-KSHV-GQ and Wt-EBV-GQ oligonucleotides were found to effectively disrupt formation of G-quadruplex structures (Fig. 3a). Native and denaturing polyacrylamide gel mobility assay indicates formation of compact intramolecular structures (under native conditions) as indicated by faster mobility compared to the mutants (Mut-KSHV-GQ and Mut-EBV-GQ) and length matched controls (Fig. 3b). The NMR spectral profiles of Wt-KSHV-GQ and Wt-EBV-GQ indicate that the imino protons linked to guanine residues involved in G-quadruplex formation exhibit a distinct chemical shift value of 10.5–12 ppm (Fig. 3c). The broad NMR resonance peaks are believed to be due to the presence of conformationally diverse G-quadruplexes undergoing conformational exchange at µs-ms timescale^92^.

As shown in Fig. 3d, DMS footprinting sequencing analysis of the FAM labelled wild type KSHV-GQ and EBV-GQ oligonucleotides reveals that the guanine repeats involved in G-quadruplex structures were protected from DMS methylation and hence not cleaved by piperidine whereas the guanine residues present in the loops of these GQs are sensitive to DMS methylation. Based on CD spectroscopy, native and denaturing PAGE, NMR spectroscopy and DMS footprinting analyses we conclude that PQS motifs found in KSHV KS-Bcl-2 and EBV BHRF1 promoters form stable intramolecular G-quadruplex structures in vitro*.*

**Figure 4:**
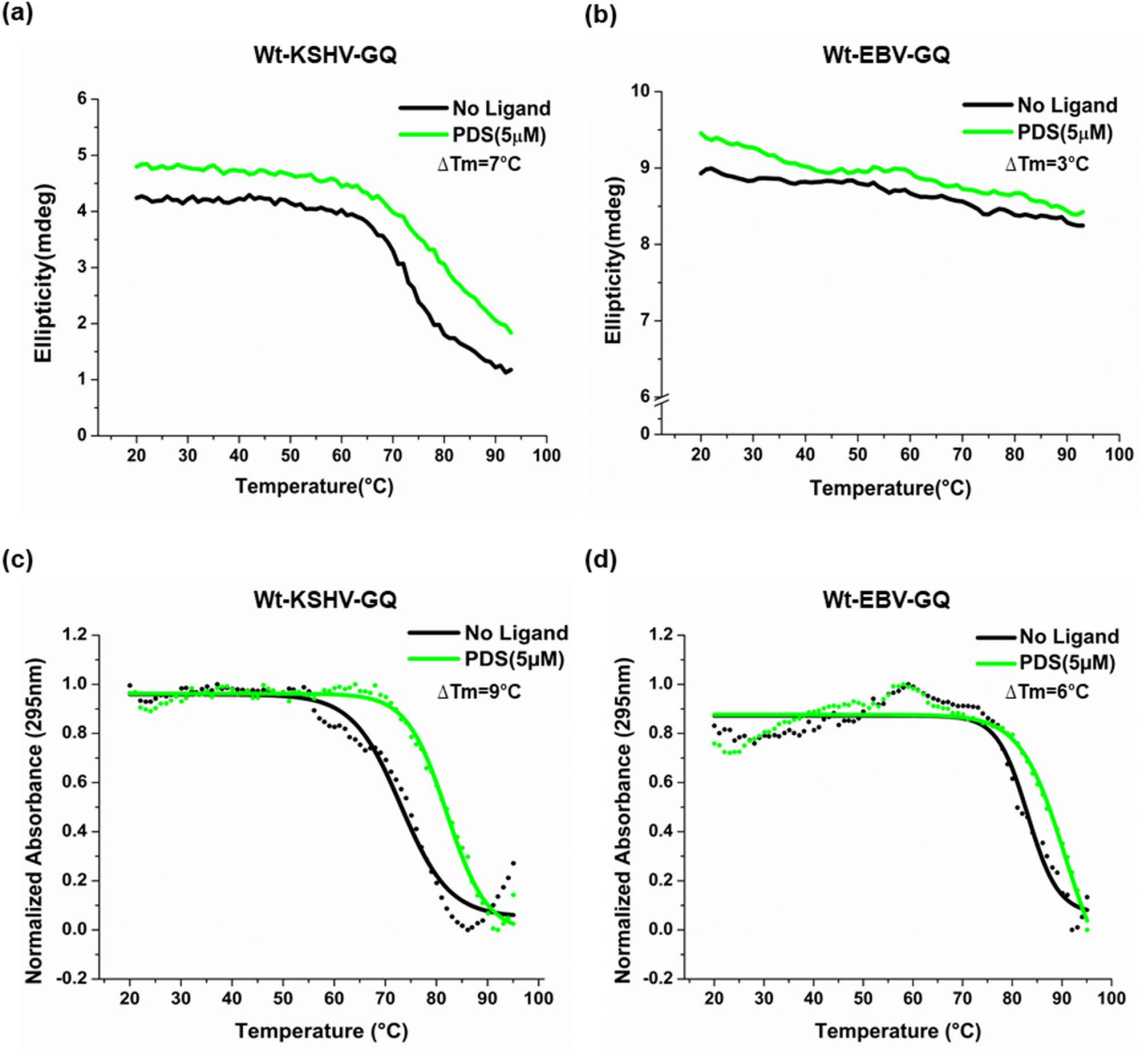
CD and UV melting studies reveal PDS-mediated stabilization of KSHV-GQ and EBV-GQ oligonucleotides. Melting analyses (20–93 °C) was performed on Wt-KSHV-GQ and Wt-EBV-GQ oligonucleotides with and without PDS (G-qu adruplex binding ligand) (**a**–**d**). As observed, PDS stabilized both KSHV-GQ and EBV-GQ. ΔT_m_ is defined as the difference between the T_m_ (melting temperature) of the PQS oligonucleotide in the presence and absence of ligand.

It is widely known that pyridostatin (PDS) (G-quadruplex ligand) specifically bind to and stabilize GQs via intercalation^27,85,93^. CD and UV melting studies with Wt-KSHV-GQ and Wt-EBV-GQ with PDS indicate PDS-mediated stabilization of KSHV-GQ and the EBV-GQ attributed to significant differences in T_m_ (melting temperature) in the presence and absence of PDS (Fig. 4a–d).

### Stabilization of G-quadruplexes in the human *bcl-2* gene promoter inhibits promoter activity

**Figure 5:**
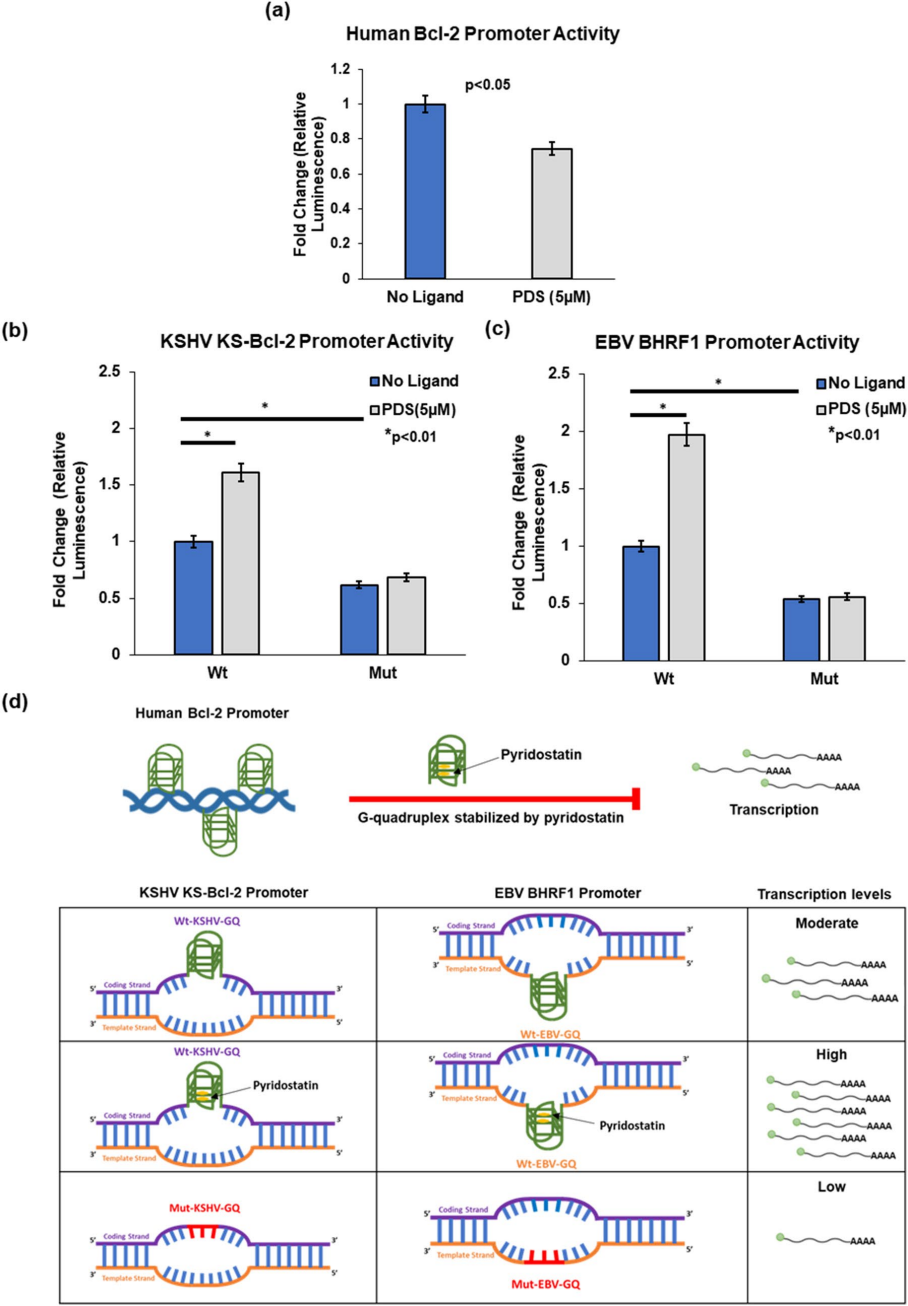
Contrasting roles for promoter G-quadruplexes in human Bcl-2 and viral Bcl-2 expression. Bar graphs show promoter activity as estimated by measuring firefly luciferase levels normalized with renilla luciferase levels (transfection control). The relative luciferase units (RLU) values of the mutant constructs (Mut-KSHV-GQ and Mut-EBV-GQ) were normalized to that of the respective wild-type constructs (Wt-KSHV-GQ and Wt-EBV-GQ). (**a**) Previous studies have established that G-quadruplexes are found in abundance in human *bcl-2* gene promoter and hence regulate its expression. Our luciferase reporter assay data is in conformity with this previously known fact. We observed that the human Bcl-2 promoter activity was significantly reduced upon exposure to PDS (5 µM). (**b**) The Wt-KSHV-GQ construct (with an intact G-quadruplex) has significantly higher promoter activity compared to that of the Mut-KSHV-GQ construct (possesses mutations disrupting G-quadruplex in the KS-Bcl-2 promoter). The addition of PDS (5 μM) led to a significant increase in promoter activity of the Wt-KSHV-GQ promoter. (**c**) The Wt-EBV-GQ construct (wild-type EBV BHRF1 promoter with an intact G-quadruplex) also exhibit higher promoter activity compared to that of the Mut-EBV-GQ construct (possesses mutations disrupting G-quadruplex in the BHRF1 promoter). We also observed that addition of PDS (5 μM) increased the promoter activity of Wt-EBV-GQ construct significantly. It is also clear from (**b**,**c**) that exposure to PDS does not alter the promoter activity the mutant reporter constructs i.e., Mut-KSHV-GQ and Mut-EBV-GQ; both containing G-quadruplex disrupting mutations). Data are depicted as mean ± SD with n = 4 replicates. (**d**) Our findings are summarized in a pictorial representation highlighting that G-quadruplexes present upstream of viral Bcl-2 homologues (i.e., KS-Bcl-2 and BHRF1) exert a positive regulatory effect on promoter activity. This finding is in contrast to the negative regulatory role for the promoter G-quadruplexes in human Bcl-2 that has been previously reported and reconfirmed under the same conditions we tested the viral Bcl-2 promoters.

Several studies have reported the presence and enrichment of G-quadruplexes within the promoter region of the human *bcl-2* gene^26,32,83^ and their stabilization by quadruplex-binding ligands further inhibits human *bcl-2* gene promoter activity^27^. Though this work is primarily aimed at understanding the role of G-quadruplexes in viral *bcl-2* gene promoters, we analysed the activity of human *bcl-2* gene promoter under similar conditions specifically used to study viral *bcl-2* gene promoter activity. We carried out luciferase-based reporter assays to assess the promoter activity of human *bcl-2* gene promoter using a luciferase construct where the human *bcl-2* gene regulatory region (-3934 bp to ATG; includes both P1 and P2 promoters) are cloned upstream of firefly luciferase coding gene of pGL3 basic vector (details are provided in “Materials and Methods” section). In agreement with previous reports^27^, we observed a significant decline in the human *bcl-2* gene promoter activity in the presence of PDS (Fig. 5a), reconfirming an inhibitory role for G-quadruplexes in the promoter.

### G-quadruplexes in the promoters of KS-Bcl-2 and BHRF1 enhance transcription

To assess the role of KSHV-GQ and EBV-GQ on promoter activity, KS-Bcl-2 and BHRF1 promoters were cloned into a promoter-less luciferase vector (pGL3 basic). We transfected these constructs (Wt-KSHV-GQ and Wt-EBV-GQ) into HEK293T cells and with varying concentrations of PDS (added 2 h post transfection). The promoter activity of the Wt-KSHV-GQ and Wt-EBV-GQ constructs (containing an intact GQ) progressively increased with increasing concentrations of PDS (0–5 µM; Supplementary File 1, Figure S1F5) in a dose-dependent manner.

Mutants were constructed to disrupt GQ formation as described in the "Material and methods" section. Luciferase reporter assays were performed using firefly luciferase constructs corresponding to KS-Bcl-2 and BHRF1 promoters (wild type and mutant); Renilla luciferase constructs (pRL-TK vector) were used as transfection controls. We observed a significantly reduced promoter activity for the Mut-KSHV-GQ and Mut-EBV-GQ constructs (with mutations disrupting GQ formation) compared to their respective wildtype promoters (Fig. 5b , c; p < 0.01). This finding suggests that the G-quadruplex motif in the KS-Bcl-2 and the BHRF1 promoter enhances promoter activity. Furthermore, the addition of PDS significantly enhanced the promoter activity of the wild-type viral *bcl-2* gene promoters (Wt-KSHV-GQ and Wt-EBV-GQ) (p < 0.01), while the promoter activity of the mutant constructs (Mut-KSHV-GQ and Mut-EBV-GQ) remained unaffected (Fig. 5b, c). This finding clearly suggests that the enhanced promoter activity in the wild-type constructs (Wt-KSHV-GQ and Wt-EBV-GQ) on addition of the ligand is due to secondary structure (G-quadruplex) stabilization and is not linked to the differences in the primary sequence.

Further, we also cloned the coding region of KS-Bcl-2 and BHRF1 genes along with their respective promoter regions (1 kb) and transfected them into HEK293T cells. The addition of PDS resulted in a significant increase in both KS-Bcl-2 and BHRF1 transcript levels as measured by real time quantitative PCR (Supplementary File 1; Figure S1F6); p < 0.01). These results strengthen our findings in reporter assays and confirm a positive regulatory role for the G-quadruplexes in the promoters of KSHV KS-Bcl-2 and EBV BHRF1.

Together, our findings suggest that the G-quadruplexes in human *bcl-2* gene promoter supress transcription, while those in viral *bcl-2* gene promoters (KS-Bcl-2 and BHRF1) enhance transcription from the respective viral *bcl-2* genes. Human herpesviruses not only captured human homologues but seem to have co-evolved to mimic secondary structures present in the promoters of their human counterparts. Interestingly, our results highlight how human herpesviruses exploit the G-quadruplexes in promoters of these homologues to regulate the expression of vBcl-2 in a manner opposite to that of the human *bcl-2* gene (Fig. 5a–d).

### G-quadruplexes in viral Bcl-2 promoters may enhance RTA-mediated induction of promoter activity

**Figure 6:**
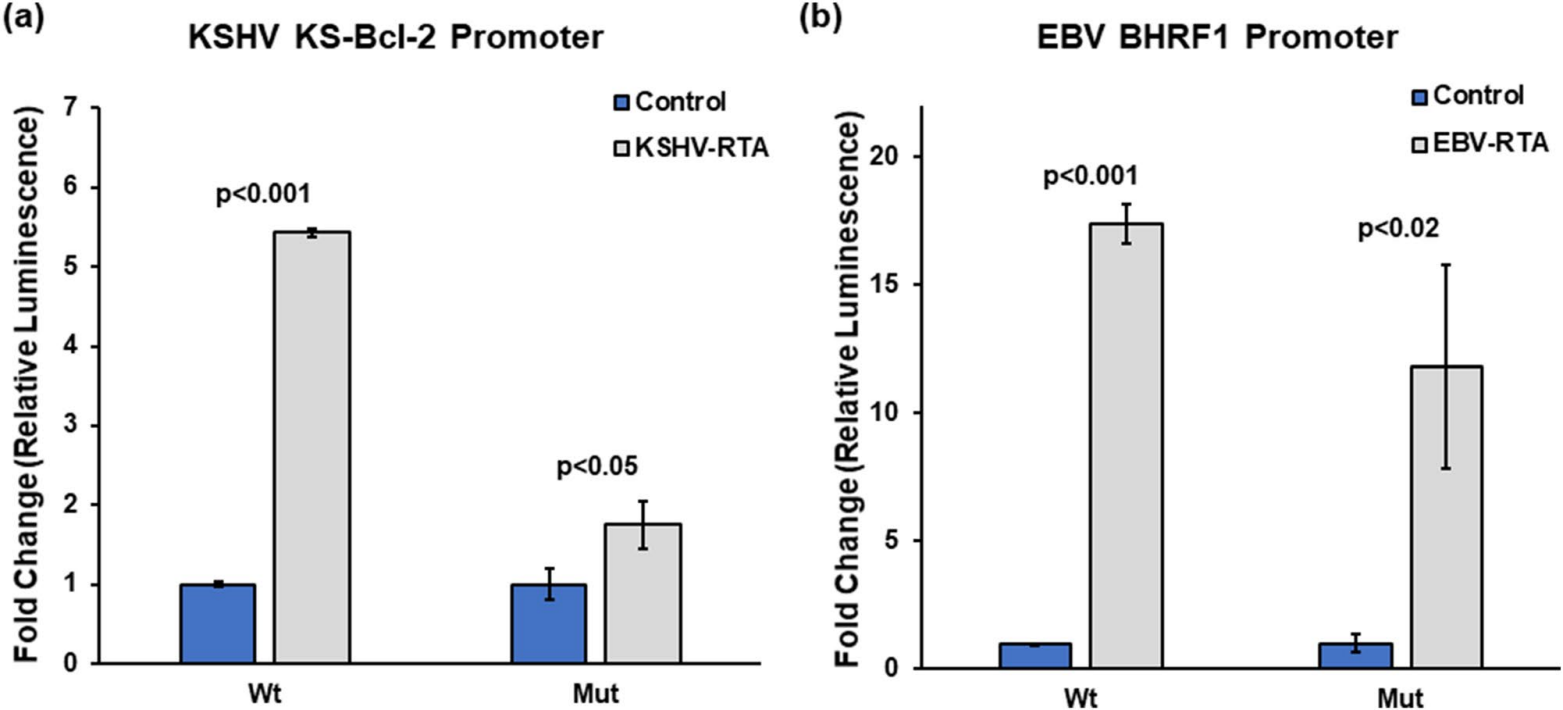
KSHV-GQ and EBV-GQ contribute to RTA induced expression of viral Bcl-2. Promoter activity of (**a**) KSHV KS-Bcl-2 and (**b**) EBV BHRF1 promoters were greatly enhanced upon co-transfection of KSHV RTA and EBV RTA, respectively. While the promoter activity of mutant promoters (with disrupted GQs) also increased significantly in the presence of viral RTAs, the extent (or fold-change) of RTA-mediated enhancement of promoter activity in the wild-type promoters (i.e., KSHV KS-Bcl-2 and EBV BHRF1) were more pronounced. Experiments were performed in triplicates and mean values ± SD are plotted.

To understand the interplay between virus-encoded transcription factors and the G-quadruplexes in viral *bcl-2* gene promoters, we chose to analyse the effect of RTAs (Replication and Transcription Activator). Viral RTAs are involved in enhancing early gene transcription during lytic replication^94–97^. KSHV RTA/ORF50 protein encoded by *orf50* gene is predominantly expressed during the lytic phase of KSHV infection and is a master regulator of several viral as well as host genes^66,94,98^. Similarly, EBV *brlf1* gene encodes for EBV RTA/BRLF1 protein which is associated with promoting lytic replication in EBV infected B cells^96,99^. RTA responsive elements (RREs) have also been reported in KSHV KS-Bcl-2 and BHRF1 gene promoters^95,100–102^. Hence, we studied the role of G-quadruplexes in KS-Bcl-2 and BHRF1 promoters in facilitating RTA-mediated regulation of viral Bcl-2 expression. KSHV RTA and EBV RTA expression constructs were co-transfected with wild type (with intact GQ) and mutant (with mutations disrupting the GQ) luciferase promoter constructs of KS-Bcl-2 and BHRF1, respectively. As expected, viral RTA enhanced promoter activity of KS-Bcl-2 and BHRF1 by several fold (Fig. 6a , b). RTA-mediated induction of the KS-Bcl-2 and the BHRF1 promoters occurred in both the wildtype (with an intact GQ) and mutant promoters (with mutations disrupting the GQ). Nonetheless, the extent of induction was more pronounced in wild-type promoters compared to mutant promoters (for both KSHV KS-Bcl-2 and EBV BHRF1; Fig. 6a, b) indicating a role for G-quadruplexes in contributing to RTA-mediated enhancement of viral Bcl-2 expression in human herpesviruses. This finding has implications beyond the regulation of vBcl-2 expression, as it provides the groundwork for further studies on analysing the role of viral RTA in regulating expression of other herpesvirus genes with promoter G-quadruplexes.

## Discussion

We identified that a sizable proportion of human herpesvirus homologues in alpha- and gamma- herpesviruses and their human counterparts have one or more G-quadruplexes in their promoters (Fig. 1 and Table 1). We did not observe this phenomenon in most of the betaherpesvirus-encoded homologues, indicating a possible role for cellular tropisms of these viruses in the acquisition of G-quadruplexes in the promoters of these homologues. The evolutionary ability of human herpesviruses to capture host genes particularly involved in critical cellular process during reactivation is well-documented^12,103–105^. Our findings suggest that when human herpesviruses acquire these host genes, they also frequently acquire G-quadruplexes which are critical regulatory elements from host gene promoters. Recent studies have pointed to the positive correlation between PQS frequency and localization in virus genomes and their corresponding hosts^34,106^. These viral PQS motifs, particularly PQS motifs found in herpesvirus genomes also share similar loop composition as their host^107,108^. This might occur due to the long-term relationship shared between the host and members of herpesviridae family that cause persistent infections in the host. It is postulated that this phenomenon might markedly influence the presence and conservation of quadruplex forming sequences in herpesvirus genomes. Hence, the resultant evolutionary accumulation of PQS motifs in viral genomes can be attributed to the mimicking of host genome organisation. We wanted to understand the biological role of these G-quadruplexes in the promoters of virus homologues. For this purpose, we chose to study the G-quadruplexes in viral Bcl-2 homologues, as they represent one of the most extensively studied herpesvirus homologues. In addition, the human *bcl-2* gene promoter is known to contain highly stable G-quadruplexes that supress transcription^27,91^. Majority of the viral Bcl-2 homologues (9 of 14 vBcl-2 homologues known till date) have promoters that contain a highly conserved G-quadruplex motif (Supplementary File 1; Figure S1F4), suggesting a potential biological role for these DNA secondary structures in regulating vBcl-2 across virus families.

Functional studies to understand the role of G-quadruplexes in the promoters of viral *bcl-2* genes were carried out on KSHV KS-Bcl-2 and EBV BHRF1. Biophysical characterization of the G-quadruplexes in KS-Bcl-2 and BHRF1 promoters using CD spectroscopy, native PAGE, NMR spectroscopy and DMS foot printing confirmed the formation of intramolecular G-quadruplex (Fig. 3). The addition of PDS stabilized both KS-Bcl-2 GQ and the BHRF1 GQ (Fig. 4). The stabilization of G-quadruplexes in the KS-Bcl-2 and BHRF1 promoters with PDS resulted in a significant increase in promoter activity (Fig. 5b,c). Disruption of the promoter G-quadruplex in KS-Bcl-2 and BHRF1 abrogated the G-quadruplex-mediated enhancement of promoter activity, and it also rendered the promoters non-responsive to the addition of PDS (Fig. 5b,c). In addition, to reporter assays, we confirmed the promoter G-quadruplex-mediated enhancement of vBcl-2 transcription in expression constructs KS-Bcl-2 and BHRF1 (with their respective native G-quadruplex containing promoter) using qPCR. Together, these findings confirm that promoter G-quadruplex in KS-Bcl-2 and BHRF1 significantly enhance vBcl-2 expression. We also reconfirmed previous reports that the G-quadruplexes in the human *bcl-2* promoters inhibit gene expression.

Altogether, we demonstrate that when human herpesviruses acquire host genes, they may also co-acquire G-quadruplexes as regulatory elements in their promoters. Nonetheless, these viruses may exploit the regulatory role of G-quadruplexes to their advantage as evidenced by diametrically opposite roles for these nucleic acid secondary structures in regulating the expression of human Bcl-2 and vBcl-2. The G-quadruplexes in the human *bcl-2* promoter inhibit the expression of this anti-apoptotic protein, thus facilitating apoptosis. It is well known that vBcl-2 homologues function similar to human Bcl-2 to inhibit apoptosis^53^. It has been reported that the position of the G-quadruplex within the regulatory element and the loop length may impact gene expression (107, 109–111). This may explain at least in part, the contrasting roles of G-quadruplexes in human and viral Bcl-2 promoters.

**Figure 7:**
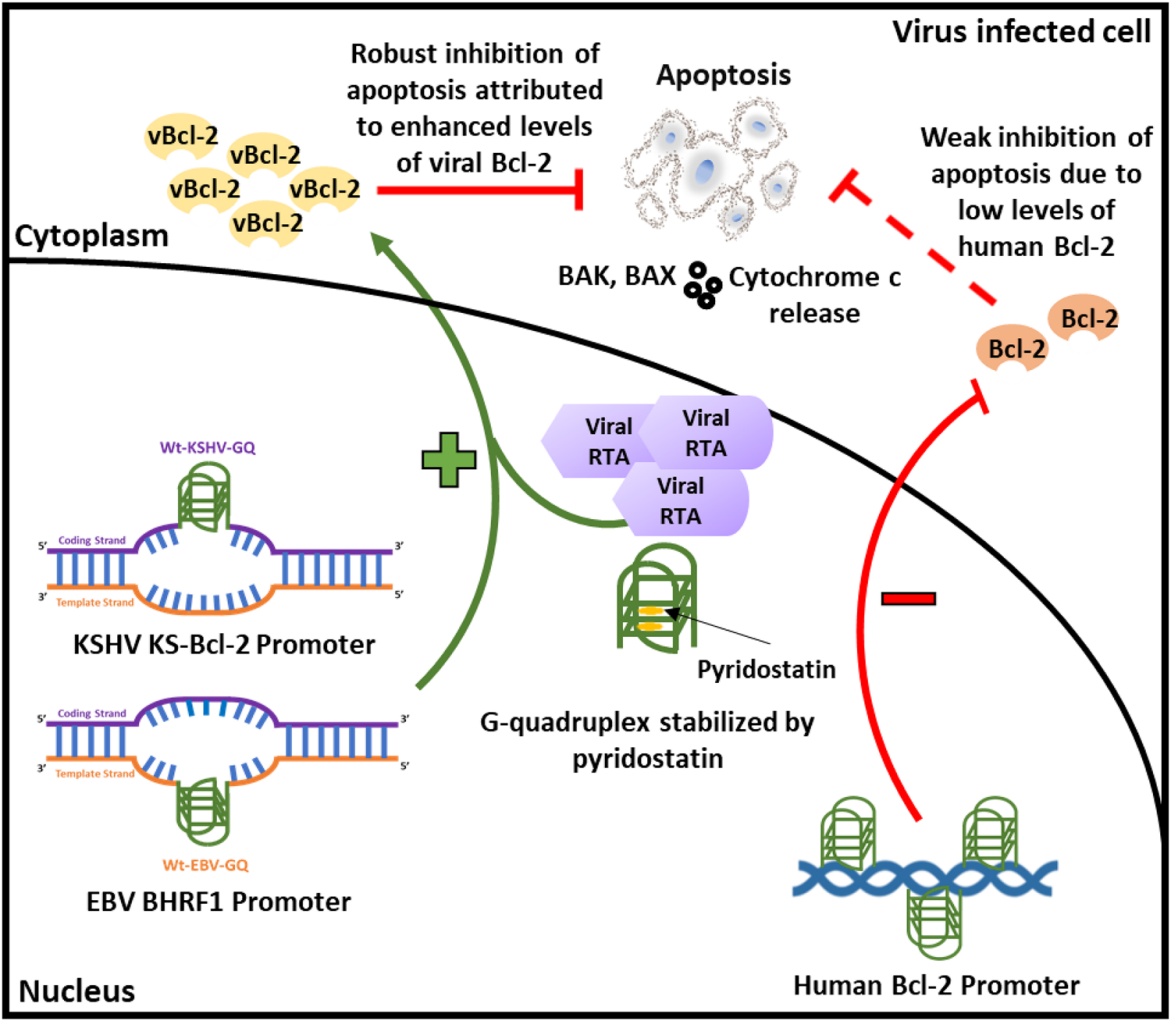
G-quadruplex-mediated regulation of viral and human Bcl-2 expression and its potential effect on host cell apoptosis. The G-quadruplexes in human Bcl-2 promoters inhibit gene expression; this may promote apoptosis of the host cell. The G-quadruplexes in viral Bcl-2 (vBcl-2) promoters serve to enhance vBcl-2 expression. Further, the G-q uadruplexes in vBcl-2 promoters contribute to RTA mediated enhancement of promoter activity. Thus, G-quadruplexes in vBcl-2 promoters enhance vBcl-2 expression which can potentially inhibit host cell apoptosis and thereby confer survival advantage to the virus.

The promoter G-quadruplex-mediated increase in the expression of vBcl-2 is particularly interesting, as it may inhibit premature death of virus infected host cells, thus facilitating virus replication, and virus survival. Furthermore, RTA, a virus-encoded transcription factor augments promoter G-quadruplex-mediated enhancement of vBcl-2 expression (Fig. 7).

In the present study, we identify the presence of G-quadruplexes in human herpesvirus homologues and their human counterparts. We demonstrate how these DNA secondary structures play opposing regulatory roles in modulation of gene expression of a given virus homologue and its human counterpart. This work highlights yet unknown complex roles for G-quadruplexes in the biology of human herpesviruses and advances our current understanding of herpesvirus pathogenesis, survival mechanisms and virus-host interactions.

## Supplementary Information

Supplementary Information 1.

Supplementary Information 2.

Supplementary Information 3.

## Data Availability

The datasets used and/or analysed during the current study are available from the corresponding author on reasonable request.

## References

[1] Mettenleiter TC, Klupp BG, Granzow H (2009). Herpesvirus assembly: An update. Virus Res..

[2] Rajčáni J, Kúdelová M (2003). Gamma herpesviruses: Pathogenesis of infection and cell signaling. Folia Microbiol. (Praha).

[3] Boehmer PE, Nimonkar AV (2003). Herpes virus replication. IUBMB Life.

[4] Feldman ER, Tibbetts SA (2015). Emerging roles of herpesvirus microRNAs during in vivo infection and pathogenesis. Curr. Pathobiol. Rep..

[5] Umbach JL, Nagel MA, Cohrs RJ, Gilden DH, Cullen BR (2009). Analysis of human alphaherpesvirus microRNA expression in latently infected human trigeminal ganglia. J. Virol..

[6] Grey F (2019). Role of microRNAs in herpesvirus latency and persistence. Virology.

[7] Chaston TB, Lidbury BA (2001). Genetic ‘budget’ of viruses and the cost to the infected host: A theory on the relationship between the genetic capacity of viruses, immune evasion, persistence and disease. Immunol. Cell Biol..

[8] McGeoch DJ, Rixon FJ, Davison AJ (2006). Topics in herpesvirus genomics and evolution. Virus Res..

[9] Davison AJ (2002). Evolution of the herpesviruses. Vet. Microbiol..

[10] Dennehy JJ (2017). Evolutionary ecology of virus emergence. Ann. N. Y. Acad. Sci..

[11] Shackelton LA, Holmes EC (2004). The evolution of large DNA viruses: Combining genomic information of viruses and their hosts. Trends Microbiol..

[12] Nicholas J (2000). Evolutionary aspects of oncogenic herpesviruses. J. Clin. Pathol. Mol. Pathol..

[13] Holzerlandt R, Orengo C, Kellam P, Mar Albà M (2002). Identification of new herpesvirus gene homologs in the human genome. Genome Res..

[14] McSharry BP, Avdic S, Slobedman B (2012). Human cytomegalovirus encoded homologs of cytokines, chemokines and their receptors: Roles in immunomodulation. Viruses.

[15] Benko M, Lenhartová S, Kempová V, Betáková T, Kúdelová M (2020). Chemokine-binding proteins encoded by herpesviruses. Acta Virol..

[16] Kvansakul M, Hinds MG (2013). Structural biology of the Bcl-2 family and its mimicry by viral proteins. Cell Death Dis..

[17] Mark L, Lee WH, Spiller OB, Proctor D, Blackbourn DJ, Villoutreix BO, Blom AM (2004). The Kaposi’s sarcoma-associated herpesvirus complement control protein mimics human molecular mechanisms for inhibition of the complement system. J. Biol. Chem..

[18] Alcami A (2003). Viral mimicry of cytokines, chemokines and their receptors. Nat. Rev. Immunol..

[19] Holzerlandt R, Orengo C, Kellam P, Alba MM (2002). Identification of new herpesvirus gene homologs in the human genome. Genome Res.

[20] Kawaguchi Y, Kato K (2003). Protein kinases conserved in herpesviruses potentially share a function mimicking the cellular protein kinase cdc2. Rev. Med. Virol..

[21] Skalsky RL, Samols MA, Plaisance KB, Boss IW, Riva A, Lopez MC, Baker HV, Renne R (2007). Kaposi’s sarcoma-associated herpesvirus encodes an ortholog of miR-155. J. Virol..

[22] Beyond Viral Interferon Regulatory Factors: Immune Evasion Strategies (2019) **29**, 1873–1881. 10.4014/jmb.1910.10004 31650769

[23] Simonsson T (2001). G-Quadruplex DNA structures—variations on a theme. Biol. Chem..

[24] Bidzinska J, Cimino-reale G, Zaffaroni N, Folini M (2013). G-quadruplex structures in the human genome as novel therapeutic targets. Molecules.

[25] Cogoi S, Xodo LE (2006). G-quadruplex formation within the promoter of the KRAS proto-oncogene and its effect on transcription. Nucleic Acids Res..

[26] Cheng Y, Tang Q, Li Y, Zhang Y, Zhao C, Yan J, You H (2019). Folding/unfolding kinetics of G-quadruplexes upstream of the P1 promoter of the human BCL-2 oncogene. J. Biol. Chem..

[27] Feng Y, Yang D, Chen H, Cheng W, Wang L, Sun H, Tang Y (2016). Stabilization of G-quadruplex DNA and inhibition of Bcl-2 expression by a pyridostatin analog. Bioorganic Med. Chem. Lett..

[28] Dai J, Dexheimer TS, Chen D, Carver M, Ambrus A, Jones RA, Yang D (2006). An intramolecular G-quadruplex structure with mixed parallel/antiparallel G-strands formed in the human BCL-2 promoter region in solution. J. Am. Chem. Soc..

[29] Yan J, Zhao D, Dong L, Pan S, Hao F, Guan Y (2017). A novel G-quadruplex motif in the Human MET promoter region. Biosci. Rep..

[30] Sun D, Guo K, Shin YJ (2011). Evidence of the formation of G-quadruplex structures in the promoter region of the human vascular endothelial growth factor gene. Nucleic Acids Res..

[31] Agrawal P, Hatzakis E, Guo K, Carver M, Yang D (2013). Solution structure of the major G-quadruplex formed in the human VEGF promoter in K +: Insights into loop interactions of the parallel G-quadruplexes. Nucleic Acids Res..

[32] Agrawal P, Lin C, Mathad RI, Carver M, Yang D (2014). The major G-quadruplex formed in the human BCL-2 proximal promoter adopts a parallel structure with a 13-nt loop in k+ solution. J. Am. Chem. Soc..

[33] Metifiot M, Amrane S, Litvak S, Andreola ML (2014). Survey and summary G-quadruplexes in viruses: Function and potential therapeutic applications. Nucleic Acids Res..

[34] Lavezzo E, Berselli M, Frasson I, Perrone R, Palù G, Brazzale AR, Richter SN, Toppo S (2018). G-quadruplex forming sequences in the genome of all known human viruses: A comprehensive guide. PLoS Comput. Biol..

[35] Saranathan N, Vivekanandan P (2019). G-quadruplexes: more than just a kink in microbial genomes. Trends Microbiol..

[36] Saranathan N, Biswas B, Patra A, Vivekanandan P (2019). G-quadruplexes may determine the landscape of recombination in HSV-1. BMC Genom..

[37] Pannecouque C, Richter SN (2014). Anti-HIV-1 activity of the G-quadruplex ligand BRACO-19 ` 1. Nucleic Acids Res.

[38] Majee P, Pattnaik A, Sahoo BR, Shankar U, Pattnaik AK, Kumar A, Nayak D (2021). Inhibition of Zika virus replication by G-quadruplex-binding ligands. Mol. Ther. Nucleic Acids.

[39] Artusi S, Nadai M, Perrone R, Angela M, Palù G, Flamand L, Calistri A, Richter SN (2015). The herpes simplex virus-1 genome contains multiple clusters of repeated G-quadruplex: Implications for the antiviral activity of a G-quadruplex ligand. Antiviral Res..

[40] Artusi S, Ruggiero E, Nadai M, Tosoni B, Perrone R, Ferino A, Zanin I, Xodo L, Flamand L, Richter SN (2021). Antiviral activity of the G-quadruplex ligand TMPyP4 against herpes simplex virus-1. Viruses.

[41] Butovskaya E, Soldà P, Scalabrin M, Nadai M, Richter SN (2019). HIV-1 nucleocapsid protein unfolds stable RNA G-quadruplexes in the viral genome and is inhibited by G-quadruplex ligands. ACS Infect. Dis..

[42] Frasson I, Soldà P, Nadai M, Lago S, Richter SN (2021). Parallel G-quadruplexes recruit the HSV-1 transcription factor ICP4 to promote viral transcription in herpes virus-infected human cells. Commun. Biol..

[43] Murat P, Zhong J, Lekieffre L, Cowieson NP, Clancy JL, Preiss T, Balasubramanian S, Khanna R, Tellam J (2014). G-quadruplexes regulate Epstein-Barr virus-encoded nuclear antigen 1 mRNA translation. Nat. Chem. Biol..

[44] Biswas B, Kandpal M, Jauhari UK, Vivekanandan P (2016). Genome-wide analysis of G-quadruplexes in herpesvirus genomes. BMC Genomics.

[45] Frasson I, Nadai M, Richter SN (2019). Conserved G-quadruplexes regulate the immediate early promoters of human alphaherpesviruses. Molecules.

[46] Ravichandran S, Kim YE, Bansal V, Ghosh A, Hur J, Subramani VK, Pradhan S, Lee MK, Kim KK, Ahn JH (2018). Genome-wide analysis of regulatory G-quadruplexes affecting gene expression in human cytomegalovirus. PLoS Pathog..

[47] Biswas B, Kandpal M, Vivekanandan P (2017). A G-quadruplex motif in an envelope gene promoter regulates transcription and virion secretion in HBV genotype B. Nucleic Acids Res..

[48] Vilmen G, Glon D, Siracusano G, Lussignol M, Shao Z, Hernandez E, Perdiz D, Quignon F, Mouna L, Poüs C (2020). BHRF1, a BCL2 viral homolog, disturbs mitochondrial dynamics and stimulates mitophagy to dampen type I IFN induction. Autophagy.

[49] Hardwick JM, Bellows DS (2003). Viral versus cellular BCL-2 proteins. Cell Death Differ.

[50] Ha J, Won E, Yoon S, Kang S, Bae K, Park SG (2009). Molecular Interaction between a Bcl-2 Homolog from Kaposi Sarcoma Virus and p53. Bull. Korea Chem. Soc..

[51] Gelgor A, Kalt I, Bergson S, Brulois KF, Jung JU, Sarid R (2015). Viral Bcl-2 encoded by the Kaposi’s sarcoma-associated herpesvirus is vital for virus reactivation. J. Virol..

[52] Watanabe A, Maruo S, Ito T, Ito M, Katsumura KR, Takada K (2010). Epstein-barr virus-encoded Bcl-2 homologue functions as a survival factor in Wp-restricted burkitt lymphoma cell line P3HR-1. J. Virol..

[53] Cuconati A, White E (2002). Viral homologs of BCL-2: Role of apoptosis in the regulation of virus infection. Genes Dev..

[54] Dreos R, Ambrosini G, Groux R, Perier RC, Bucher P (2017). The eukaryotic promoter database in its 30th year: Focus on non-vertebrate organisms. Nucleic Acids Res..

[55] Nandakumar D, Glaunsinger B (2019). An integrative approach identifies direct targets of the late viral transcription complex and an expanded promoter recognition motif in Kaposi’s sarcoma-associated herpesvirus. PLoS Pathog..

[56] Rossetto CC, Tarrant-Elorza M, Verma S, Purushothaman P, Pari GS (2013). Regulation of viral and cellular gene expression by Kaposi’s sarcoma-associated herpesvirus polyadenylated nuclear RNA. J. Virol..

[57] Palmeri D, Spadavecchia S, Carroll KD, Lukac DM (2007). Promoter- and cell-specific transcriptional transactivation by the kaposi’s sarcoma-associated herpesvirus ORF57/Mta protein. J. Virol..

[58] Matsuura M, Takemoto M, Yamanishi K, Mori Y (2011). Human herpesvirus 6 major immediate early promoter has strong activity in T cells and is useful for heterologous gene expression. Virol. J..

[59] Haque M, Davis DA, Wang V, Widmer I, Yarchoan R (2003). Kaposi’s sarcoma-associated herpesvirus (human herpesvirus 8) contains hypoxia response elements: Relevance to lytic induction by hypoxia. J. Virol..

[60] Pickett BE, Sadat EL, Zhang Y, Noronha JM, Squires RB, Hunt V, Liu M, Kumar S, Zaremba S, Gu Z (2012). ViPR: An open bioinformatics database and analysis resource for virology research. Nucleic Acids Res..

[61] Huppert JL, Balasubramanian S (2005). Prevalence of quadruplexes in the human genome. Nucleic Acids Res..

[62] Kumar S, Choudhary D, Patra A, Bhavesh NS, Vivekanandan P (2020). Analysis of G-quadruplexes upstream of herpesvirus miRNAs: Evidence of G-quadruplex mediated regulation of KSHV miR-K12-1-9,11 cluster and HCMV miR-US33. BMC Mol. cell Biol..

[63] Jiang M, Anderson J, Gillespie J, Mayne M (2008). uShuffle : A useful tool for shuffling biological sequences while preserving the k-let counts. BMC Bioinform..

[64] Xie G (2013). 乳鼠心肌提取 HHS public access. Physiol. Behav..

[65] Heckman CA, Mehew JW, Ying GG, Introna M, Golay J, Boxer LM (2000). A-Myb up-regulates bcl-2 through a Cdx binding site in t(14;18) lymphoma cells. J. Biol. Chem..

[66] Gao J, Cai Q, Lu J, Jha HC, Robertson ES (2011). Upregulation of cellular Bcl-2 by the KSHV encoded RTA promotes virion production. PLoS ONE.

[67] Sarisky RT, Gao Z, Lieberman PM, Fixman ED, Hayward GS, Hayward SD (1996). A replication function associated with the activation domain of the Epstein-Barr virus Zta transactivator. J. Virol..

[68] Rao X, Huang X, Zhou Z, Lin X (2013). An improvement of the 2ˆ(-delta delta CT) method for quantitative real-time polymerase chain reaction data analysis. Biostat. Bioinforma. Biomath..

[69] Huppert JL, Balasubramanian S (2007). G-quadruplexes in promoters throughout the human genome. Nucleic Acids Res..

[70] Davis ZH, Verschueren E, Jang GM, Kleffman K, Johnson JR, Park J, VonDollen J, Maher MC, Johnson T, Newton W (2015). Global mapping of herpesvirus-host protein complexes reveals a transcription strategy for late genes. Mol. Cell.

[71] Li J, Liu W, Che K, Zhang Y, Zhao Z, Luo B (2017). (2017) The methylation status and expression of Epstein-barr virus early genes BARF1 and BHRF1 in Epstein-barr virus-associated gastric carcinomas. Gastroenterol. Res. Pract..

[72] Matsuura M, Takemoto M, Yamanishi K, Mori Y (2011). Human herpesvirus 6 major immediate early promoter has strong activity in T cells and is useful for heterologous gene expression. Virol. J..

[73] Ruvolo PP, Deng X, May WS (2001). Phosphorylation of Bcl2 and regulation of apoptosis. Leukemia.

[74] Cory S, Adams JM (2002). The BCL2 family: Regulators of the cellular life-or-death switch. Nat. Rev. Cancer.

[75] Han J, Sabbatini P, Perez D, Rao L, Modha D, White E (1996). The E1B 19K protein blocks apoptosis by interacting with and inhibiting the p53-inducible and death-promoting Bax protein. Genes Dev..

[76] Boyd JM, Malstrom S, Subramanian T, Venkatesh LK, Schaeper U, Elangovan B, D’Sa-Eipper C, Chinnadurai G (1994). Adenovirus E1B 19 kDa and Bcl-2 proteins interact with a common set of cellular proteins. Cell.

[77] Lin PW, Huang YJ, John JAC, Chang YN, Yuan CH, Chen WY, Yeh CH, Shen ST, Lin FP, Tsui WH (2008). Iridovirus Bcl-2 protein inhibits apoptosis in the early stage of viral infection. Apoptosis.

[78] Kvansakul M, Caria S, Hinds MG (2017). The Bcl-2 family in host-virus interactions. Viruses.

[79] De Laval VR, Deléage G, Aouacheria A, Combet C (2014). BCL2DB: Database of BCL-2 family members and BH3-only proteins. Database.

[80] Cheng EHY, Nicholas J, Bellows DS, Hayward GS, Guo HG, Reitz MS, Hardwick JM (1997). A Bcl-2 homolog encoded by Kaposi sarcoma-associated virus, human herpesvirus 8, inhibits apoptosis but does not heterodimerize with Bax or Bak. Proc. Natl. Acad. Sci. U. S. A..

[81] Oudejans JJ, Van Den Brule AJC, Jiwa NM, De Bruin PC, Ossenkoppele GJ, Van Der Valk P, Walboomers JMM, Meijer CJLM (1995). BHRF1, the Epstein-Barr virus (EBV) homologue of the BCL-2 proto-oncogene, is transcribed in EBV-associated B-cell lymphomas and in reactive lymphocytes. Blood.

[82] Nambiar M, Goldsmith G, Moorthy BT, Lieber MR (2010). Formation of a G-quadruplex at the BCL2 major breakpoint region of the t (14; 18) translocation in follicular lymphoma. Nucleic Acids Res..

[83] Dai J, Chen D, Jones RA, Hurley LH, Yang D (2006). NMR solution structure of the major G-quadruplex structure formed in the human BCL2 promoter region. Nucleic Acids Res..

[84] Zhou W, Suntharalingam K, Brand NJ, Barton PJR, Vilar R, Ying L (2013). Possible regulatory roles of promoter G-quadruplexes in cardiac function-related genes - Human TnIc as a model. PLoS ONE.

[85] Kong JN, Zhang C, Zhu YC, Zhong K, Wang J, Chu BB, Yang GY (2018). Identification and characterization of G-quadruplex formation within the EP0 promoter of pseudorabies virus. Sci. Rep..

[86] Huppert JL, Bugaut A, Kumari S, Balasubramanian S (2008). G-quadruplexes: The beginning and end of UTRs. Nucleic Acids Res..

[87] Verma A, Halder K, Halder R, Yadav VK, Rawal P, Thakur RK, Mohd F (2008). Genome-wide computational and expression analyses reveal g-quadruplex DNA motifs as conserved cis-regulatory elements in human and related species. J. Med. Chem..

[88] Zhao Y, Du Z, Li N (2007). Extensive selection for the enrichment of G4 DNA motifs in transcriptional regulatory regions of warm blooded animals. Febs Lett..

[89] Brooks TA, Hurley LH (2010). Targeting MYC expression through G-quadruplexes. Genes Cancer.

[90] Fleming AM, Zhu J, Ding Y, Burrows CJ (2019). Location dependence of the transcriptional response of a potential G-quadruplex in gene promoters under oxidative stress. Nucleic Acids Res..

[91] Avi A, National S, Gonz C, National S (2017). Targeting the G-quadruplex-forming region near the P1 promoter in the human BCL-2 gene with the cationic porphyrin TMPy. J. Biol. Chem..

[92] Harkness RW, Mittermaier AK (2017). G-quadruplex dynamics. Biochim. Biophys. Acta Proteins Proteomics.

[93] Manchon JFM, Koellhoffer EC, Gopakumar J, Kim N, Mccullough LD, Tsvetkov AS (2017). The G-quadruplex DNA stabilizing drug pyridostatin promotes DNA damage and downregulates transcription of Brca1 in neurons. Aging.

[94] Bellare P, Ganem D (2009). Regulation of KSHV lytic switch protein expression by a virus-encoded microRNA: an evolutionary adaptation that fine-tunes lytic reactivation. Cell Host Microbe.

[95] Chang LK, Chung JY, Hong YR, Ichimura T, Nakao M, Liu ST (2005). Activation of Sp1-mediated transcription by Rta of Epstein-Barr virus via an interaction with MCAF1. Nucleic Acids Res..

[96] El-Guindy A, Ghiassi-Nejad M, Golden S, Delecluse H-J, Miller G (2013). Essential role of Rta in lytic DNA replication of epstein-barr virus. J. Virol..

[97] AuCoin DP, Colletti KS, Cei SA, Papousková I, Tarrant M, Pari GS (2004). Amplification of the Kaposi’s sarcoma-associated herpesvirus/human herpesvirus 8 lytic origin of DNA replication is dependent upon a cis-acting AT-rich region and an ORF50 response element and the trans-acting factors ORF50 (K-Rta) and K8 (K-bZIP). Virology.

[98] West JT, Wood C (2003). The role of Kaposi’s sarcoma-associated herpesvirus/human herpesvirus-8 regulator of transcription activation (RTA) in control of gene expression. Oncogene.

[99] Ragoczy T, Miller G (1999). Role of the epstein-barr virus RTA protein in activation of distinct classes of viral lytic cycle genes. J. Virol..

[100] Campbell M, Izumiya Y (2020). PAN RNA: Transcriptional exhaust from a viral engine. J. Biomed. Sci..

[101] Papp B, Motlagh N, Smindak RJ, Jin Jang S, Sharma A, Alonso JD, Toth Z (2018). Genome-wide identification of direct RTA targets reveals key host factors for kaposi’s sarcoma-associated herpesvirus lytic reactivation. J. Virol..

[102] Heilmann AMF, Calderwood MA, Portal D, Lu Y, Johannsen E (2012). Genome-wide analysis of epstein-barr virus Rta DNA binding. J. Virol..

[103] Fu M, Deng R, Wang J, Wang X (2008). Detection and analysis of horizontal gene transfer in herpesvirus. Virus Res..

[104] Schönrich G, Abdelaziz MO, Raftery MJ (2017). Herpesviral capture of immunomodulatory host genes. Virus Genes.

[105] Raftery M, Muller A, Schonrich G (2000). Herpesvirus homologues of cellular genes. Virus Genes.

[106] Bohálová N, Cantara A, Bartas M, Kaura P, Štastný J, Pečinka P, Fojta M, Brázda V (2021). Tracing dsdna virus–host coevolution through correlation of their g-quadruplex-forming sequences. Int. J. Mol. Sci..

[107] Lombardi EP, Londoño-Vallejo A, Nicolas A (2019). Relationship between G-quadruplex sequence composition in viruses and their hosts. Molecules.

[108] Bohálová N, Cantara A, Bartas M, Kaura P, Šťastný J, Pečinka P, Fojta M, Mergny JL, Brázda V (2021). Analyses of viral genomes for G-quadruplex forming sequences reveal their correlation with the type of infection. Biochimie.

[109] Holder IT, Hartig JS (2014). A matter of location: Influence of G-quadruplexes on escherichia coli gene expression. Chem. Biol..

[110] Lim KW, Jenjaroenpun P, Low ZJ, Khong ZJ, Ng YS, Kuznetsov VA, Tu A (2015). Duplex stem-loop-containing quadruplex motifs in the human genome: a combined genomic and structural study. Nucleic Acid Res..

[111] Lightfoot HL, Hagen T, Tatum NJ, Hall J (2019). The diverse structural landscape of quadruplexes. FEBS Lett..

