# Supplementary material for: Contrasting roles for G-quadruplexes in regulating human Bcl-2 and virus homologues KSHV KS-Bcl-2 and EBV BHRF1
Source: Sci Rep. 2022 Mar 23;12:5019. doi: 10.1038/s41598-022-08161-9 (PMC8943185; doi:10.1038/s41598-022-08161-9)
Supplement: Supplementary file 1 — Supplementary Information 1. [file 41598_2022_8161_MOESM1_ESM.pdf]

Supplementary File 1, Table S1T1

List of human genes (along with their PQS densities) captured by human herpesviruses.

Gene	Full Name	Ensemble ID	Length (base pair)	Orientation	PQS/kb (-5000bp-+100bp)	
					Coding Strand	Template Strand
**POLA**	Polymerase (DNA directed) alpha	ENSG00000101868	303068	+	0	0.392156863
**HFM1**	DNA Helicase	ENSG00000162669	144104	-	0.392156863	0.196078431
**UNG1**	Uracil DNA glycosylase	ENSG00000076248	13419	+	0.196078431	0.196078431
**RRM1**	Ribonucleotide reductase M1 polypeptide	ENSG00000167325	44170	+	0.196078431	0
**RRM2**	Ribonucleotide reductase M2 polypeptide	ENSG00000171848	9092	+	0.392156863	0.196078431
**TYMS**	Thymiylate synthetase	ENSG00000176890	15975	+	0.196078431	0
**DHFR**	Dihydrofolate reductase	ENSG00000228716	28756	-	0.392156863	0.196078431
**PCTK2**	Protein kinase cdc2-related PICTAIRE-2	ENSG00000059758	122300	-	0.196078431	0.588235294
**PRP4**	Serine/threonine-protein kinase PRP4	ENSG00000112739	43717	+	0	0
**RFP**	Ring finger protein (C3H2C3 type) 6	ENSG00000127870	90379	-	0	0
**OX-2**	OX-2 membrane glycoprotein precursor	ENSG00000091972	30465	+	0	0
**FEN-1**	Flap structure–specific endonuclease 1	ENSG00000168496	4608	+	0.392156863	0.784313725
**CKR2**	Chemokine (C–C motif) receptor 2	ENSG00000121807	7195	+	0	0
**GPR50**	G protein coupled receptor 50	ENSG00000102195	4813	+	0.392156863	0
**TNFRSF14**	Tumor necrosis factor receptor superfamily member 14	ENSG00000273936	9984	+	0.784313725	0.196078431
**IP-9**	Small inducible cytokine subf. B, member 9B	ENSG00000169248	7733	-	0	0
**TSC-1**	Small inducible cytokine subf. A, member 26	ENSG00000006606	20363	-	0.588235294	0.392156863
**HLA1-E**	Major histocompatibility complex, class I, E	ENSG00000229252	4739	+	0.196078431	0
**IL10**	Interleukin 10	ENSG00000136634	4893	-	0.196078431	0
**BCL2**	B–cell lymphoma protein 2	ENSG00000171791	196783	-	0.980392157	1.176470588
**CD80**	CD80 antigen	ENSG00000121594	35310	-	0	0
**ICSBP1**	Interferon consensus seq. binding prot. 1	ENSG00000140968	23807	+	0	0.392156863
**IRF4**	Interferon regulatory factor 4	ENSG00000137265	19709	+	0.196078431	0.392156863
**IL6**	Interleukin 6	ENSG00000136244	6119	+	0.588235294	0.392156863
**DAF**	Decay accelerating factor for complement	ENSG00000196352	65297	+	0.196078431	0.392156863
**CCND1**	Cyclin D1	ENSG00000110092	13388	+	1.568627451	0.784313725
**HLA1**	Major histocompatibility complex, class I	ENSG00000234487	3062	+	0.392156863	0
**FLIP**	CASP8 and FADD–like apoptosis regulator	ENSG00000003402	60584	+	0	0.196078431

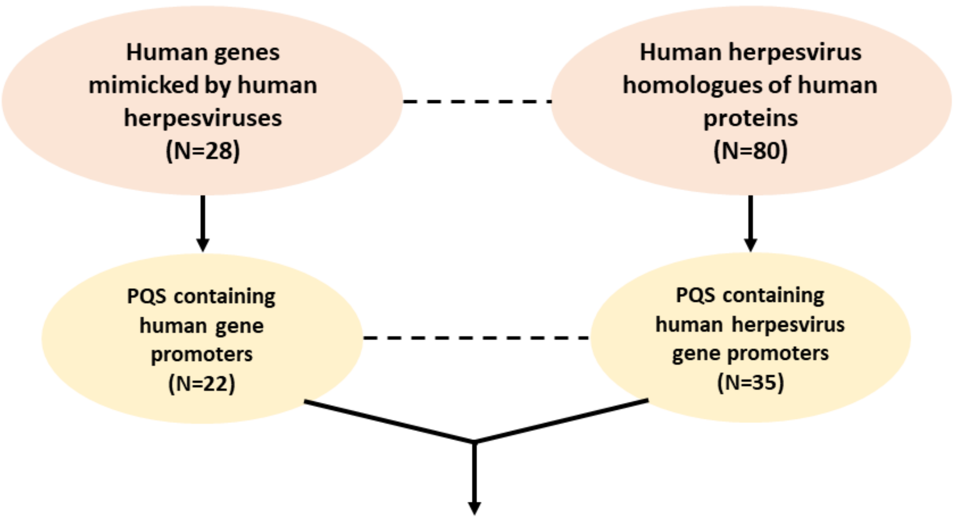

PQS containing promoters of herpesvirus homologues and their human counterparts

Human Genes	Human Herpesvirus Homologues
DNA polymerase alpha (POLA)	1-UL30, 2-UL30
DNA helicase (HFM1)	1-UL5, 2-UL5
Uracil DNA glycosylase (UNG1)	1-UL2, 2-UL2, 4-BKRF3
Ribonucleotide reductase M1 polypeptide (RRM1)	1-UL39, 2-UL39, 4-BORF2
Ribonucleotide reductase M2 polypeptide (RRM2)	1-UL40, 2-UL40, 4-BaRF1
Protein kinase cdc2-related PICTAIRE-2 (PCTK2)	2-UL3, 8-ORF36
Flap structure–specific endonuclease 1 (FEN1)	1-UL41, 2-UL41
G protein-coupled receptor 50 (GPR50)	8-ORF74
Interleukin 10 (IL10)	4-BCRF1
B-cell lymphoma protein 2 (Bcl-2)	4-BHRF1, 4-BALF1, 8-KS-Bcl-2
Interferon consensus seq. binding protein 1 (ICSBP1)	8-vIRF1
Interferon regulatory protein 4 (IRF4)	8-vIRF3
Interleukin 6 (IL6)	8-K2
Decay accelerating factor for complement (DAF)	8-ORF4
Cyclin D1 (CCND1)	8-ORF72
Major histocompatibility complex, class I (HLA1)	8-K3, 8-K5

Supplementary File 1, Figure S1F1

PQS motifs in regulatory region of human herpesvirus homologues and their human counterparts. The list of human genes (n=16) that contain promoter G-quadruplexes along with their corresponding viral homologues (n=29) with promoter G-quadruplexes is shown.

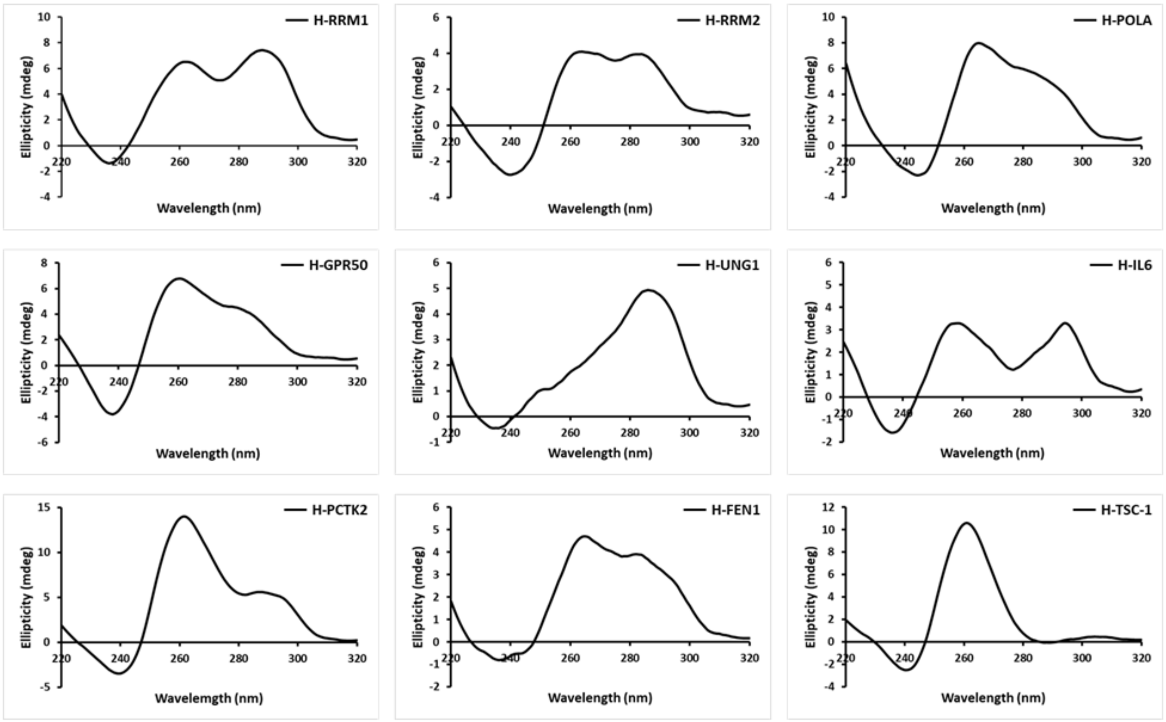

Supplementary File 1, Figure S1F2

CD spectral profiles of randomly selected PQS motifs (n=9) found in promoters of human genes that are captured by herpesviruses. The sequence of the oligonucleotides used are listed in Supplementary File 2, Table S2T3.

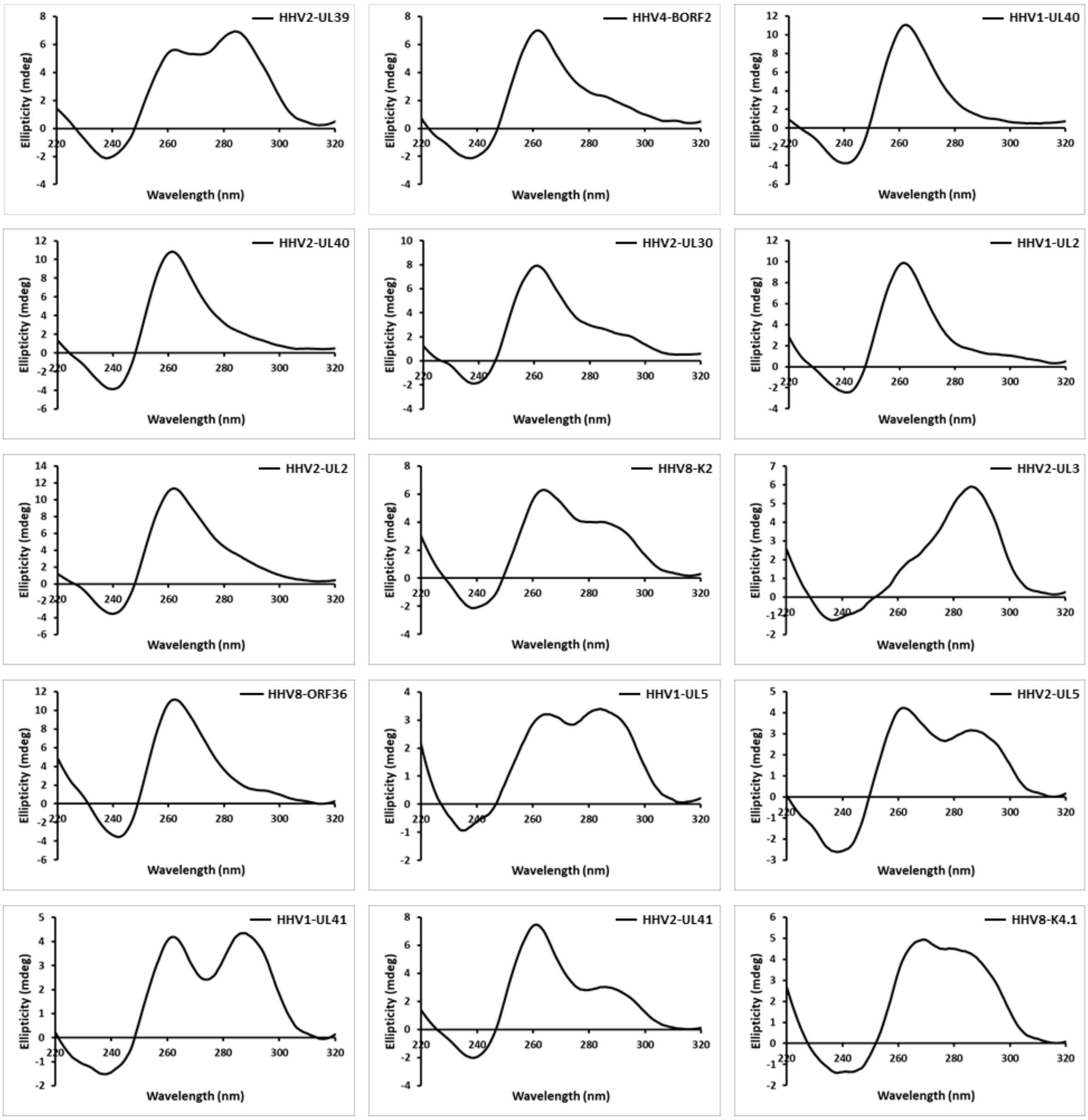

Supplementary File 1, Figure S1F3

CD spectral profiles of randomly selected PQS motifs (n=15) found in promoters of human herpesvirus homologues. The sequence of the oligonucleotides used are listed in Supplementary File 2, Table S2T4.

Supplementary File 1, Table S1T2

Viral Bcl-2 homologues from herpesviruses and other groups of viruses are enriched for PQS motifs in their upstream regulatory regions. Bcl-2 homologues from 9 viruses contained PQS motifs in the 1 kb upstream regulatory regions. All viruses known to mimic cellular *bcl-2* gene till date are listed in the table. The highlighted viruses (bold red font) possessing at least 1 PQS motif in their 1 kb upstream regulatory region. All available sequences for these viruses were considered for analysis of PQS (Details are provided in (Supplementary File 2; Table S2T2).

Viral Family	Virus
Adenoviridae	**Adenovirus**
Asfarviridae	African swine fever virus
Herpesviridae	Alcelaphine herpesvirus 1
	**Epstein Barr Virus BHRF1**
	**Epstein Barr Virus BALF1**
	Bovine herpesvirus 4
	**Murine gammaherpesvirus 68**
	**Kaposi's sarcoma associated herpesvirus**
	**Meleagrid herpesvirus 1**
Iridoviridae	Lymphocystis disease virus
	**Frog virus 3**
	**Singapore grouper iridovirus**
Poxviridae	Fowlpox virus
	**ORF virus**

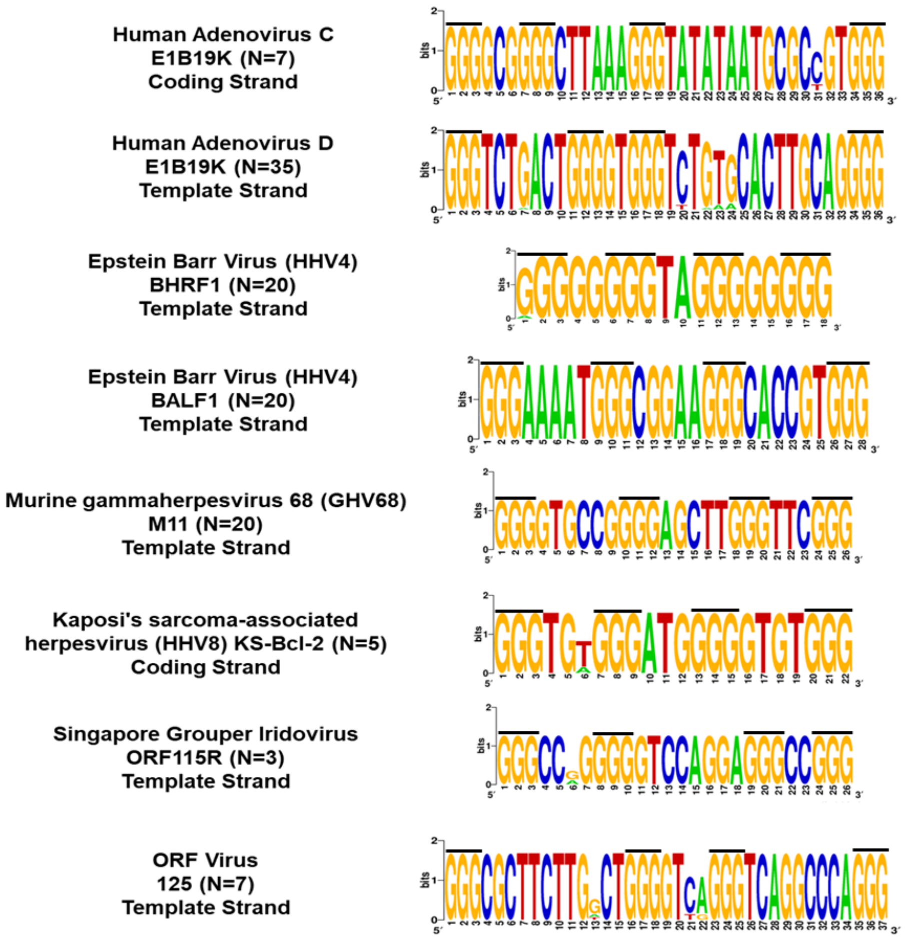

Supplementary File 1, Figure S1F4

Conservation analysis of PQS motifs found in the upstream regulatory regions of viral Bcl-2 homologues among all viruses (herpesviruses and other groups of viruses). Sequence logos of highly conserved PQS motifs found in regulatory regions of viral Bcl-2 homologues (n=8). The G residues which are a part of the G-quadruplex tetrad are marked by a black bar over the residues. N represents the number of sequences considered for PQS conservation analysis. (Please see Supplementary File 2; Table S2T2 for more details).

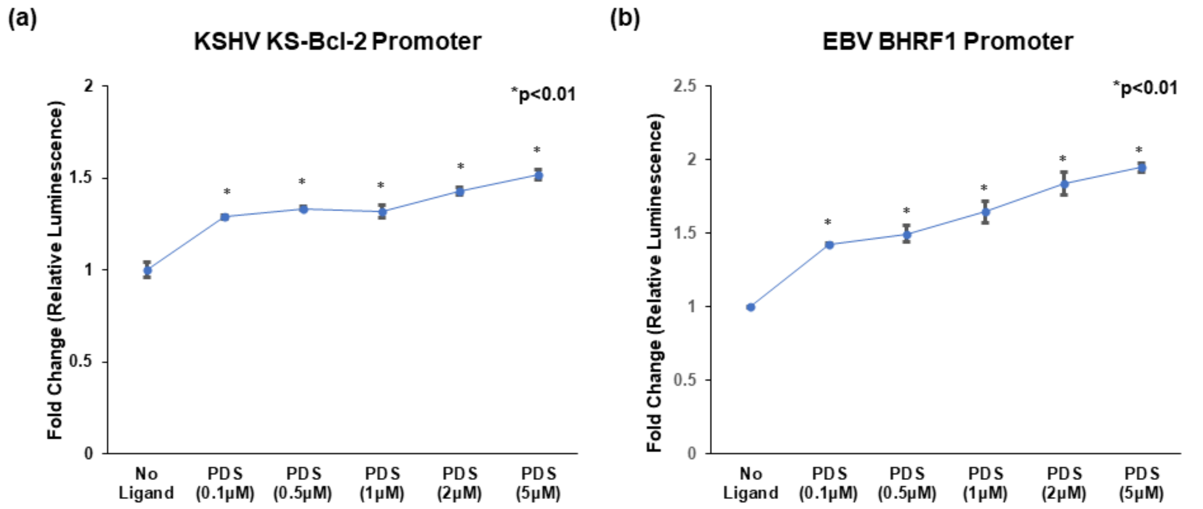

Supplementary File 1, Figure S1F5

Effect of varying doses of PDS on wild type KS-Bcl-2 and BHRF1 promoter activity. Bar graphs (a) and (b) shows dose dependent response of wild type KS-Bcl-2 and BHRF1 promoters to pyridostatin (0-5 µM) as measured by luciferase reporter assays.

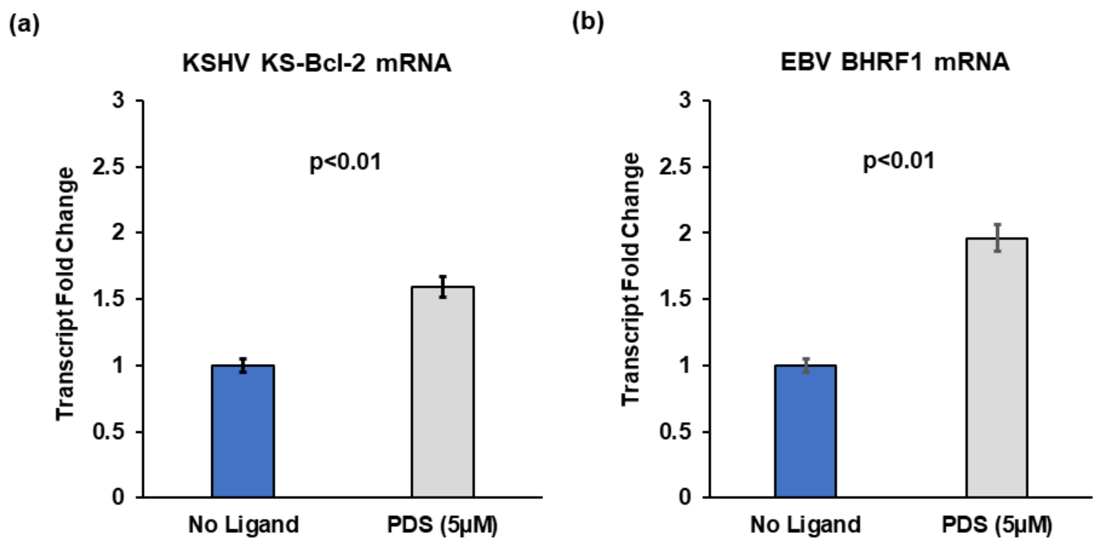

Supplementary File 1, Figure S1F6

Pyridostatin upregulates KSHV KS-Bcl-2 and EBV BHRF1 mRNA levels. Bar graphs (a) and (b) show increased expression of KS-Bcl-2 and EBV BHRF1 mRNAs from their native promoters (with intact G-quadruplexes: Wt-KSHV-GQ and Wt-EBV-GQ) on addition of PDS. The viral Bcl-2 transcripts were quantified using real time PCR using appropriate primers. Experiments were performed in triplicates and mean values ±SD are plotted.
