# Supplementary material for: Contrasting roles for G-quadruplexes in regulating human Bcl-2 and virus homologues KSHV KS-Bcl-2 and EBV BHRF1
Source: Sci Rep. 2022 Mar 23;12:5019. doi: 10.1038/s41598-022-08161-9 (PMC8943185; doi:10.1038/s41598-022-08161-9)
Supplement: Supplementary file 2 — Supplementary Information 2. [file 41598_2022_8161_MOESM2_ESM.pdf]

Supplementary File 2, Table S2T1

List of virus strains used for PQS identification.

Virus	Accession Number
**HHV1 (HSV-1)**	AB618031, FJ593289, GU734771, GU734772, JN555585, JQ673480, JQ780693, KF498959, NC_001806, X14112
**HHV2 (HSV-1)**	JN561323, NC_001798, Z86099, KF781518
**HHV3 (VZV)**	DQ479957, JN704697, JN704698, DQ479955, DQ479956, DQ479961, DQ479962, DQ479963, DQ479958, DQ479959, DQ479960, KC847290, AY548171, DQ457052, NC_001348 , JQ972913, DQ452050, AJ871403, DQ479954, JQ972914, JF306641, AY548170, DQ674250, DQ479953, EU154348, KC112914, DQ008354, DQ008355, JN704696, JN704700, JN704707, JN704690, JN704705, JN704704, JN704708, JN704702, JN704701, JN704710, JN704695, JN704692, JN704691, JN704709, JN704699, JN704703, JN704706, JN704693, JN704694, KF811485, KJ767491, KJ767492, KJ808816, X04370
**HHV4 (EBV)**	DQ279927, AJ507799, NC_007605 , V01555, AY961628, LN827563, KF717093, LN827548, AP015016, AP015015, AB850649, AB850654, AB850647, AB850643, KP735248, LN827557, KC207813, HQ020558, AB828190, LC137018
**HHV5 (HCMV)**	AC146851, AC146904, AC146905, AC146906, AC146907, AC146999, AY315197, AY446894, BK000394, EF999921, FJ527563, FJ616285, GQ121041, GQ221973, GQ221974, GQ221975, GQ396662, GQ396663, GQ466044, GU179001, GU179288, GU179289, GU179290, GU937742, GU980198, HQ380895, JX512197, JX512198, JX512199, JX512200, JX512201, JX512202, JX512203, JX512204, JX512205, JX512206, JX512207, JX512208, KF021605, KF297339, KF493876, KF493877, KJ361971, KJ426589, KM192298, KM192299, KM192300, KM192301, KM192302, NC_006273, X17403
**HHV6A**	KC465951, KJ123690, NC_001664
**HHV7**	AF037218, NC_001716
**HHV8 (KSHV)**	JQ619843, AF148805, NC_009333, GQ994935, HQ404500, KF703446, U75698.1

Supplementary File 2, Table S2T2

List of virus strains used for PQS identification and conservation.

Virus	Accession Number
**Adenovirus**	HAdV-C1 AC_000017, HAdV-C2 AC_0000073, HAdV-B3 AY599834, HAdV-E4 AY599837, HAdV-C5 AY601635, HAdV-C6 FJ349096, HAdV-B7 KP670856.2, HAdV-D8 AB448767, HAdV-D9 AJ854486, HAdV-D10 JN226746, HAdV-B11 AF532578, HAdV-A12 X73487, HAdV-D13 JN226747, HAdV-B14 JQ824845, HAdV-D15 KF268204, HAdV-B16 JN860680, HAdV-D17 HQ910407, HAdV-A18 GU191019, HAdV-D19 JQ326209, HAdV-D20 JN226749, HAdV-B21 AY601633, HAdV-D22 FJ619037, HAdV-D23 JN226750, HAdV-D24 JN226751, HAdV-D25 JN226752, HAdV-D26 EF153474, HAdV-D27 JN226753, HAdV-D28 FJ824826, HAdV-D29 JN226754, HAdV-

	D30 JN226755, HAdV-A31 AM749299, HAdV-D32 JN226756, HAdV-D33 JN226758, HAdV-B34 AY737797, HAdV-B35 AC_000019, HAdV-D36 GQ384080, HAdV-D37 AB448775, HAdV-D38 JN226759, HAdV-D39 JN226760, HAdV-F40 NC_001454, HAdV-F41 DQ315364.2, HAdV-D42 JN226761, HAdV-D43 JN226762, HAdV-D44 JN226763, HAdV-D45 JN226764, HAdV-D46 AY875648, HAdV-D47 JN226757, HAdV-D48 EF153473, HAdV-D49 DQ393829, HAdV-B50 AY737798, HAdV-D51 JN226765
**African swine fever virus**	KM262845.1, NC_044941.1, NC_044943.1, MN270969.1, MN270970.1, MN270971.1, MN270972.1, MN270973.1, MN270974.1, MN270975.1, MN270976.1, MN270977.1, MN270978.1, MN270979.1, MN270980.1, KX354450.1, NC_044955.1, U18466.2, NC_001659.2, KM262844.1, MH910495.1, MK333180.1, MK333181.1, MK645909.1, MN715134.1, MK940252.1, FR682468.2, MT459800.1, MH766894.1, MH681419.1, NC_044959.2, MN194591.1, MK128995.1, MK543947.1, KM111294.1, NC_044945.1, MH025918.1, MH025917.1, MH025916.1, MH025919.1, MH025920.1, KM111295.1, NC_044946.1, KM102979.1, NC_044947.1, LR881473.1, MN913970.1, AY261362.1, NC_044953.1, LS478113.1, MT496893.1, MT847620.1, MT847621.1, MT847622.1, MT847623.1, LR536725.1, LR722599.1, LR722600.1, MK628478.1, MN172368.1, MN393476.1, MN393477.1, MN641876.1, AY261366.1, NC_044949.1, MN641877.1, AY261364.1, NC_044951.1, MN630494.1, NC_044952.1, AY261363.1, AY261365.1, NC_044950.1, AY261361.1, NC_044954.1, AY261360.1, NC_044944.1, MN336500.2, MN394630.2, MN318203.2
**Alcelaphine Herpesvirus 1**	MG000864.1, KX905134.1, KX905135.1, KX905136.1
**Bovine herpesvirus 4**	JN133502.1
**Murine gammaherpesvirus 68**	AF105037.1, U97553.2, NC_001826
**Meleagrid herpesvirus 1**	AF282130.1, AF291866.1, NC_002641.1
**Lymphocystis disease virus**	NC_005902.1, AY380826.1
**Frog virus 3**	MF360246.1, EU627010.1, MG791866.1, JQ654586.1, KJ175144.1, KJ538546.1, MG953520.1, MG953519.1, MG953518.1, MH351268.1, MK959621.1, MK959616.1, MK959610.1, MK959609.1, MK959608.1, MK959607.1, MK959606.1, MT578298.1, KF512820.1, KC243313.1, KX574341.1, AY548484.1, KX397570.1, MK959620.1, MK959619.1, MK959618.1, MK959615.1, MK959614.1, MK959613.1, MK959611.1, KP266742.1, KP266742.1, KX185156.1, MK227779.1, MT512502.1, MT512497.1, MT512497.1, AF389451.1, MT512504.1, MT512503.1, MT512501.1, MT512500.1, MT512499.1, MT512498.1, KX574343.1, MK959612.1
**Singapore Grouper iridovirus**	AY521625.1, NC_006549.1, AY666015.1
**Fowlpox virus**	AF198100, KX196452, NC_002188, MH734528, MH719203, MH709124, MH709125, MF766431, MF766430
**ORF virus**	KF234407, KY053526, DQ184476, LR594616, AX754989, HM133903, NC_005336,

Supplementary File 2, Table S2T3

Sequence of randomly selected PQS oligonucleotides for promoter regions of human genes mimicked by human herpesviruses used for CD experiments.

Oligonucleotide Name	Sequence (5'--3')
(H)-RRM1	GGGTGAGGAGTGGTCAGGGTAGGGGCAGGG
(H)-RRM2	GGGCCACGCGGCAATGCGGGCGGTTCCGCCTCCGGGGAGGTCGGG
(H)-POLA	GGGAAAAGATTTGGGCGCGTGGGAGAAAGGG
(H)-GPR50	GGGGAGCGGGTCTGCACCCTGGGACCTAGGG
(H)-UNG1	GGGCTCAGGGGTTAATGGGTTAATGGG
(H)-IL6	GGGAGGATTCCCAAGGGGTCACTTGGGAGAGGGCAGGG
(H)-PCTK2	GGGGGCGGGGCCGGCGGCCGGGGGCGGGG
(H)-FEN1	GGGTGTAGAGGGAGCAGGGGCCTGCGGG
(H)-TSC-1	GGGAGGGAGGGAGGG

Supplementary File 2, Table S2T4

Sequence of randomly selected PQS oligonucleotides for promoter regions of human herpesvirus homologues used for CD experiments.

Oligonucleotide Name	Sequence (5'--3')
HHV2-UL39	GGGCGGTCTGGGTTACCACAACAGGTGGGTGCTTCGGG
HHV4-BORF2	GGGTGTAGCCGCCCGGGTCGAGGCGGGTGGGGG
HHV1-UL40	GGGGGTGGGTGGGCTCCAGGCACGGG
HHV2-UL40	GGGGGTGGGCGGGGTCCAGGCAAGGCAGGGCCTGGG
HHV2-UL30	GGGGCGTGGGAGGGGCTGGGG
HHV1-UL2	GGGATTTTGGGTTGGGTCGGG
HHV2-UL2	GGGCTTGTCGTTATGGGAGCCTGGGGGGCGTGGGGTGGG
HHV8-K2	GGGTCATCGGGGGTGGGAACTTGTACCGGG
HHV2-UL3	GGGTCGGGCCTGATCGCGTTCTGGGCATGGG
HHV8-ORF36	GGGTGAAAAATAACGGGGGATGGAGGAAGAGGGATGGG
HHV1-UL5	GGGCAGCTGGGGTATACCGAGGGCCAGGGGG
HHV2-UL5	GGGAGGGCGATATCGCCGGGGTGGCCGAGCGGG
HHV1-UL41	GGGCCGGGGTCTCGGGTGCGGG
HHV2-UL41	GGGCTCGGGGGAGGGGGAGGG
HHV8-K4.1	GGGAGTTCTAGGGATAGGGGCCAATGGG

Supplementary File 2, Table S2T5

Sequence of PQS oligonucleotides for promoter regions of KSHV KS-Bcl-2 and EBV BHRF1 used for biophysical experiments.

Oligonucleotide Name	Sequence (5'--3')
Wt-KSHV-GQ	GGGGTGTGGGATGGGGGTGTGGG
Mut-KSHV-GQ	AGAGTGTGAGATGAGAGTGTGAG
Control (C) 23 mer	AGAGAGAGAGAGAGAGAGAGAGA
Wt-EBV-GQ	GGGGGGGGTAGGGGGGGG

Mut-EBV-GQ	GAGAGAGATAGAGAGAGA
Control (C) 18 mer	AGAGAGAGAGAGAGAGAG

Supplementary File 2, Table S2T6

Sequence of 5' FAM labelled PQS oligonucleotides for promoter regions of KSHV KS-Bcl-2 and EBV BHRF1 used for DMS foot printing experiments.

**Oligonucleotide Name**	**Sequence (5'FAM--3')**
Wt-KSHV-GQ	TTTTTTTTTTGGGGTGTGGGATGGGGGTGTGGG
Wt-EBV-GQ	TTTTTTTTTTGGGGGGGGTAGGGGGGGG

Supplementary File 2, Table S2T7

Sequence of primer oligonucleotides used for creation of luciferase constructs.

**Luciferase Reporter Constructs**	**Sequence (5'--3')**
Wt-KSHV-GQ-luc-Fwd	AA*GGTACC*TGATATGCG AGGATACTTA
Wt-KSHV-GQ-luc-Rev	AT*AAGCTT*TTGTGGACGGTCACCAGGG
Mut-KSHV-GQ-luc-Fwd	GGA*CGTCTC*GTGTGAGATGAGAGTGTGGGATGG
Mut-KSHV-GQ-luc-Rev	GGA*CGTCTC*TCACACTCTCATCTCACACCCCCAT
Wt-EBV-GQ-luc-Fwd	AA*GAGCTC*GGTCCCCATGGCACAGGCCT
Wt-EBV-GQ-luc-Rev	AA*AAGCTT*AGGAGCCGTCCTTATTCTTGCTCATA
Mut-EBV-GQ-luc-Fwd	AT*CGTCTC*CCCGGCGAGAGTGAGGAGTG
Mut-EBV-GQ-luc-Rev	AA*CGTCTC*GCCGGGCTCCTGGT

Supplementary File 2, Table S2T8

Sequence of primer oligonucleotides used for qPCR experiments.

**Primer name**	**Sequence (5'--3')**
KS-Bcl-2-FP	AGATTTCACAGCACCACCGGTA
KS-Bcl-2-RP	CCCCAGTTCATGTTTCCATCGC
BHRF1-FP	GTACCCTGCATCCTGTGTTG
BHRF1-RP	CTACAGTGTCCTCTGGCGAA

Supplementary File 2, Table S2T9

Detailed description of PQS motifs found in putative promoter regions of homologous genes encoded by human herpesviruses.

**Virus**	**Gene**	**Coding Strand**		**Template Strand**		**Proximity to TSS**	**Overlap of PQS with other genomic features ***
		**PQS Sequence (5'-> 3')**	**PQS Location**	**PQS Sequence (5'-> 3')**	**PQS Location**		
HHV-1 (*X14112.1*)	UL30	GGGGAGGGTT GGAG ACCGGGGTTGG GG	62164..62190	GGGGCGTGGGAG GGGCTGGGG	62373..62393	501-1000 bp, 501-1000 bp	Yes
	UL5			GGGCAGCTGGGGT ATACCGAGGGCCA	15262..15292	<500 bp	Yes

				GGGGG			
	UL2	GGGATTTTGGG TTGG GTCGGG	9340..9360	GGGAGGGGCCGCG TATGGGCGGG	8965..8987	501-1000 bp, 501-1000 bp	Yes
	UL39			GGGTTTCGGCGGG CTCCAGAGGGGAC GTCGGCGGG	86394..86428	<500 bp	Yes
	UL40			GGGGGTGGGTGGG CTCCAGGCACGGG	89554..89579	<500 bp	Yes
	UL41	GGGCCGGGGT CTCG GGTGCGGG	93031..93052			<500 bp	Yes
HHV-2 (*NC_001798.2*)	UL30	GGGTGGGCGG GTGGG	63227..63242			<500 bp	Yes
	UL5			GGGAGGGCGATAT CGCCGGGGTGGCC GAGCGGG	15620..15652	<500 bp	Yes
	UL2	GGGCTTGTCG TTATGGGAGCC TGGG GGGC GTGGGGTGGG	9484--9522			<500 bp	Yes
	UL39	GGGCGGTCTG GGTT ACCACAA CAGGTGGGTG CTTCGGG	86803..86840	GGGCCCGTGGGAG GCGGGGTGGG	86989..86701	<500 bp, <500 bp	Yes
	UL40			GGGGGTGGGCGGG GTCCAGGCAAGGCA GGGCCTGGG	90175..90210	<500 bp	Yes
	UL3			GGGTCGGGCCTGAT CGCGTTCTGGGCAT GGG	10848..10878	<500 bp	Yes
	UL41	GGGCACGCCT TCGC CCCGGGG GACCGGGGAA CGCGGG	92858-92894			501-1000 bp	Yes
HHV-4 (*NC_007605.1*)	BKRF3			GGGCCAGGTTGTG GGCCGGGTCCAGG GGCCATTCCAAAG GG	97279..97319	501-1000 bp	Yes
	BORF2	GGGTGTAGCC GCC CGGGTCGAGG CGG GTGGGGG	63366..63398			501-1000 bp	No
	BaRF1	GGGCCCAGGG GAG GGACCAGGTG GGTGGTGTGT	66461..66500			<500 bp	Yes

		GCC<br>CGGG					
	BcRF1	GGGATCCGCC<br>AGG<br>GTCTGCAGGAT<br>AA<br>ACATGGGAGG<br>GG	124579..124616			501-1000 bp	No
	BHRF1			GGGGGGGGTAGG<br>GGGGGG	41096..41114	501-1000 bp	Yes
	BALF1			GGGAAAATGGGC<br>GGAAGGGCACCG<br>TGGG	165789..165816	501-1000 bp	Yes
HHV-8 (*JQ619843.1*)	ORF36			GGGTGAAAAATAA<br>CGGGGGATGGAG<br>GAAGAGGGATGGG	59013..59050	501-1000 bp	Yes
	ORF74			GGGCCGCGGGAA<br>GGTCATGGGCCA<br>AGGG	127249..127246	501-1000 bp	Yes
	KS-Bcl-2	GGGTGTGGGA<br>TGGGG<br>GTGAGGG	28931..28952			<500 bp	Yes
		GGGGTGTGGG<br>ATGG<br>GGGTGTGGG	28956..28978			<500 bp	Yes
		GGGTGGGGG<br>TGT<br>GGGATGGGG	29007..29027			<500 bp	Yes
	vIRF1	GGGTATGGT<br>GGG<br>TGGGGGTGG<br>AGGG	85583..85559			501-1000 bp	Yes
	vIRF3	GGGTGGACA<br>GTG<br>CCTGGGTGC<br>TGTCC<br>CGGGTTATG<br>GG	93193..93229			501-1000 bp	Yes
	K2			GGGCGGGGTCGC<br>GGGTCGGGG	17799..17819	<500 bp	No
	ORF4	GGGTCATCGGGG<br>GTGGGAACT<br>TGT<br>ACCGGG	18034..18054			501-1000 bp	Yes
	ORF72			GGGCGACTGGTCT<br>GGTCCAGGGTGGG<br>GCAAGCAGGACAC<br>GGG	122592..122663	<500 bp	Yes
	K3	GGGCGGATCC<br>GGAGGGAAGG<br>AAGGGCGGGG	26178..26207	GGGTTTTGGGCAC<br>GCTGGGGACGTG<br>GG	26593..26619	<500 bp	Yes

	K5			GGGCGGGGGGTGA CCCAACATAGTGAT TCGGGAGATTTGGG	27602..27642	501-1000 bp	Yes

*Genomic features include ori sites and ORF regions.
