# Supplementary material for: Contrasting roles for G-quadruplexes in regulating human Bcl-2 and virus homologues KSHV KS-Bcl-2 and EBV BHRF1
Source: Sci Rep. 2022 Mar 23;12:5019. doi: 10.1038/s41598-022-08161-9 (PMC8943185; doi:10.1038/s41598-022-08161-9)

KSHV-GQ

Native

Wt Mut C

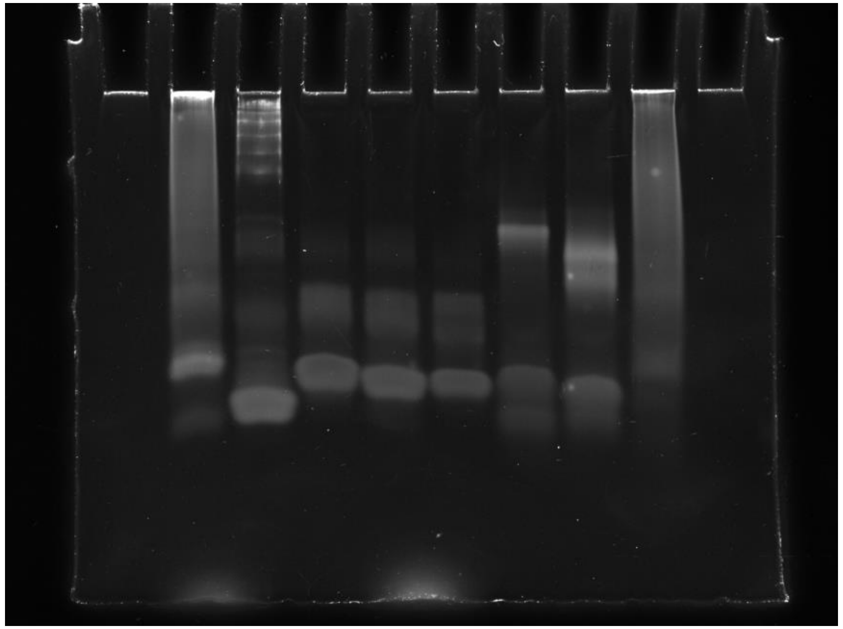

KSHV-GQ

Denaturing

Wt Mut C

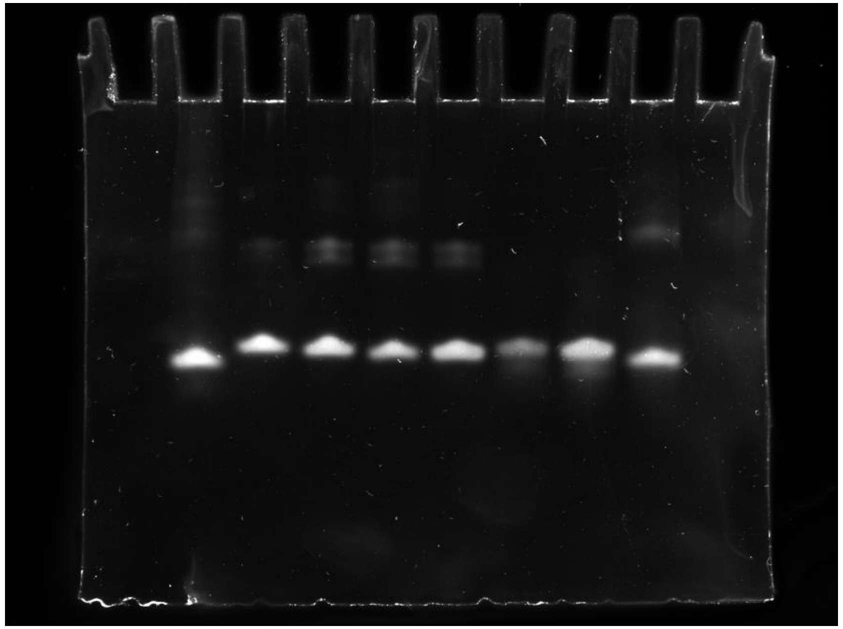

EBV-GQ
Native
Wt Mut C

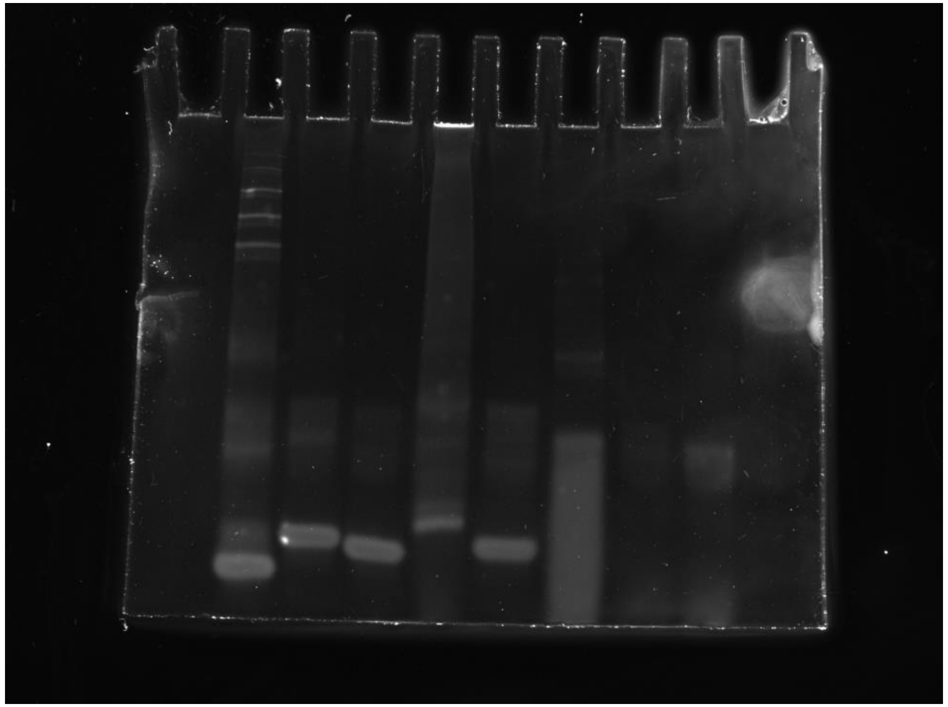

EBV-GQ
Denaturing
Wt Mut C

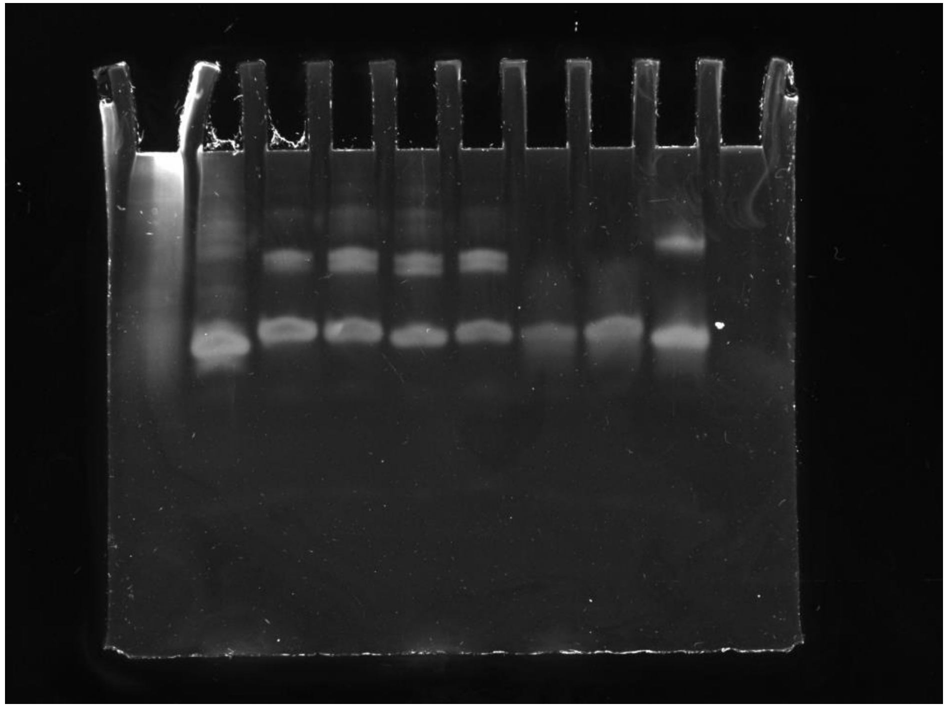

DMS Footprinting KSHV-GQ

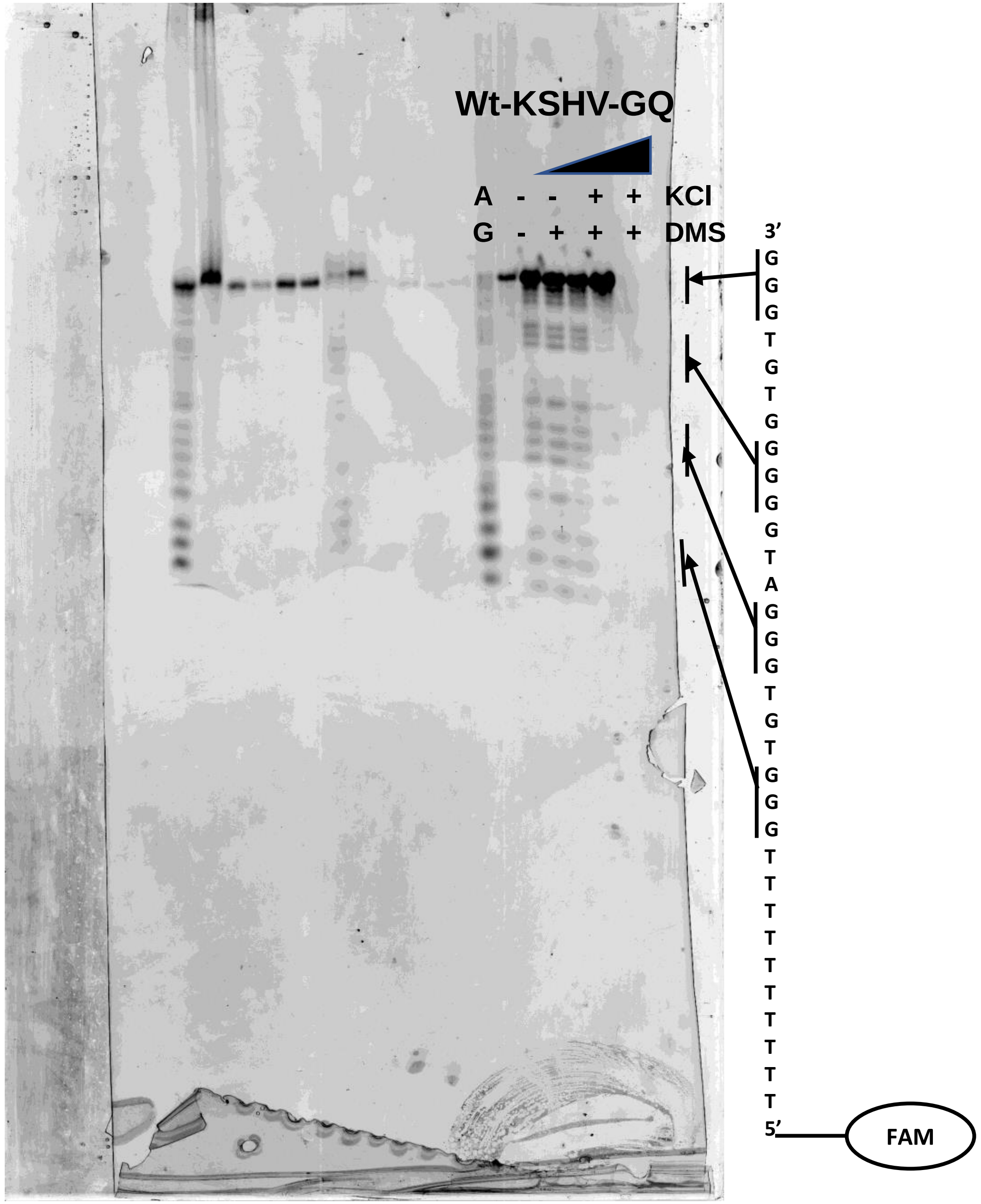

DMS
Footprinting
EBV-GQ

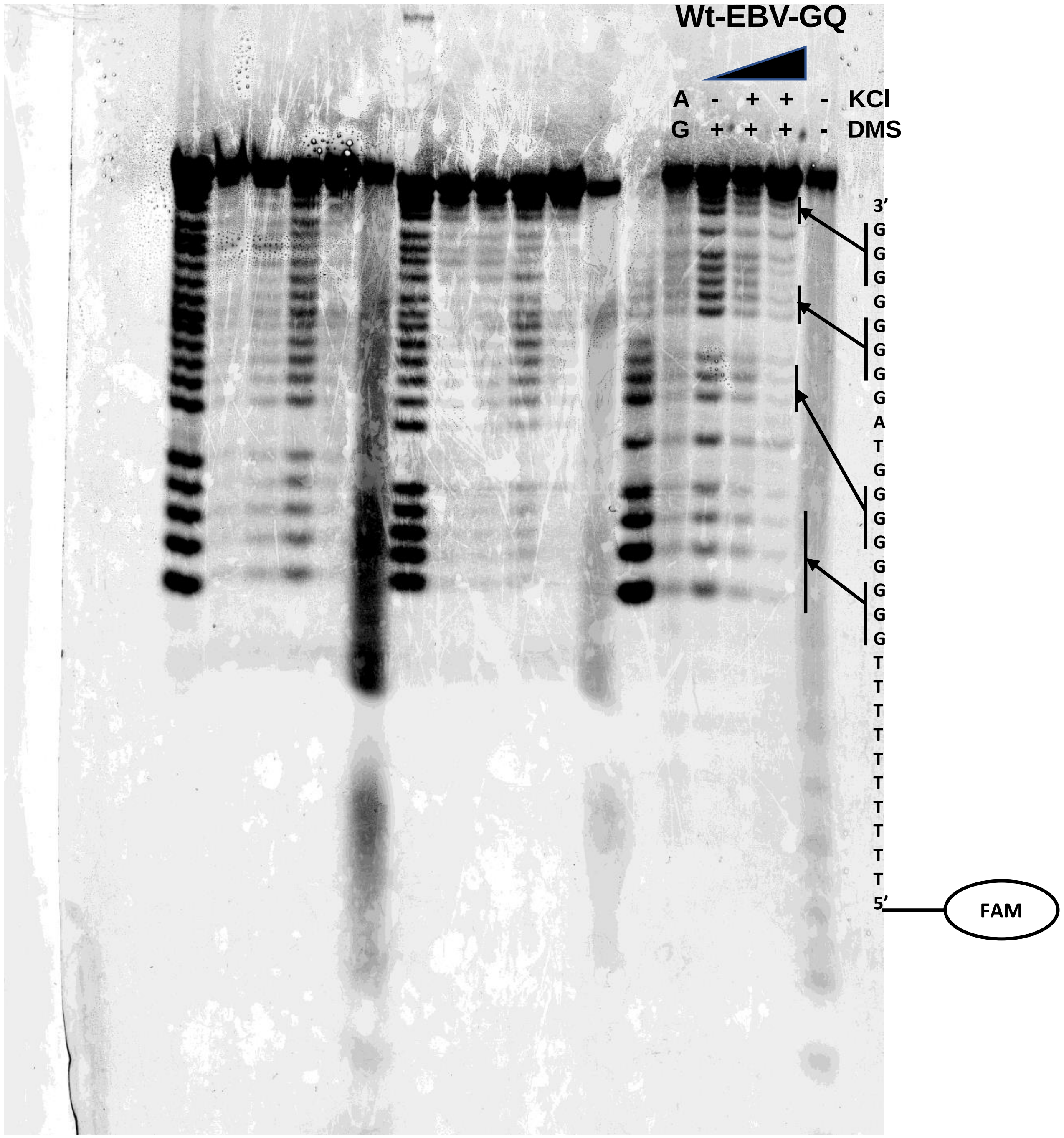

Supplement: Supplementary file 3 — Supplementary Information 3. [file 41598_2022_8161_MOESM3_ESM.pdf]
